# Multi-epitope Models Explain How Pre-existing Antibodies Affect the Generation of Broadly Protective Responses to Influenza

**DOI:** 10.1371/journal.ppat.1005692

**Published:** 2016-06-23

**Authors:** Veronika I. Zarnitsyna, Jennie Lavine, Ali Ellebedy, Rafi Ahmed, Rustom Antia

**Affiliations:** 1 Department of Microbiology and Immunology, Emory University School of Medicine, Atlanta, Georgia, United States of America; 2 Department of Biology, Emory University, Atlanta, Georgia, United States of America; 3 Emory Vaccine Center, Atlanta, Georgia, United States of America; University of Michigan, UNITED STATES

## Abstract

The development of next-generation influenza vaccines that elicit strain-transcendent immunity against both seasonal and pandemic viruses is a key public health goal. Targeting the evolutionarily conserved epitopes on the stem of influenza’s major surface molecule, hemagglutinin, is an appealing prospect, and novel vaccine formulations show promising results in animal model systems. However, studies in humans indicate that natural infection and vaccination result in limited boosting of antibodies to the stem of HA, and the level of stem-specific antibody elicited is insufficient to provide broad strain-transcendent immunity. Here, we use mathematical models of the humoral immune response to explore how pre-existing immunity affects the ability of vaccines to boost antibodies to the head and stem of HA in humans, and, in particular, how it leads to the apparent lack of boosting of broadly cross-reactive antibodies to the stem epitopes. We consider hypotheses where binding of antibody to an epitope: (i) results in more rapid clearance of the antigen; (ii) leads to the formation of antigen-antibody complexes which inhibit B cell activation through Fc*γ* receptor-mediated mechanism; and (iii) masks the epitope and prevents the stimulation and proliferation of specific B cells. We find that only epitope masking but not the former two mechanisms to be key in recapitulating patterns in data. We discuss the ramifications of our findings for the development of vaccines against both seasonal and pandemic influenza.

## Author Summary

The current influenza vaccine requires frequent updating in order to protect against small changes in the virus from one year to the next as well as larger changes associated with the emergence of new influenza strains from zoonotic reservoirs that cause pandemics. There is a considerable interest in developing “universal” vaccines that will boost immune responses to the conserved regions of the virus, in particular, to the stem region of the major virus surface molecule hemagglutinin (HA). However, recent data reveals that vaccination results in very limited boosting of antibodies to the stem of HA. We use mathematical models to explore different hypotheses that may explain why vaccination does not boost antibodies to the conserved parts of the virus. By confronting our models with the data from the human vaccination trials we found that the key mechanism preventing effective boosting of the responses to the stem of HA is masking of the stem by pre-existing antibodies developed during previous infections and vaccinations. We discuss how this masking effect could be overcome in a “universal” influenza vaccine.

## Introduction

Both seasonal and pandemic influenza pose significant public health concerns. Seasonal influenza in the U.S. is estimated to lead to an economic burden of $87.1 billion [1], and pandemic influenza poses a grave threat to public health, as witnessed during the 1918–1919 Spanish influenza outbreak [2].

We are currently able to generate vaccines against seasonal influenza based on knowledge of its global patterns of spread and mechanisms of evolution, such as antigenic drift, that lead to gradual annual changes in the surface proteins of the virus. However, given current immunization technologies, a new vaccine must be formulated each year; this endeavor is costly, and estimates of vaccine effectiveness vary widely and differ depending on whether they are focused on symptoms, infection or transmission [3–7]. Moreover, current vaccine technologies are not protective against pandemic influenza strains, to which people have little or no pre-existing humoral immunity. Pandemic influenza generally occurs due to larger antigenic changes (shifts), and when these novel strains enter the human population they typically cause severe disease [2, 8].

The humoral immune response is most strongly stimulated by hemagglutinin (HA), the major surface molecule, which has a distinct head and stem structure [9–12]. Current influenza vaccines target the head of hemagglutinin, which has multiple epitopes that vary from year to year. In contrast, the stem is highly conserved and remains largely the same over time. In fact, there are only a couple of different stem types, even across influenza subtypes. The stem is therefore a desirable target for immunization because a vaccine that could elicit antibodies that bind to the stem epitopes would be useful across years and even, likely, for novel pandemic strains that may arise in the future.

Recent experimental studies in mice and ferrets show that it is possible to generate high levels of antibody to the stem of HA using novel vaccines, and these antibodies can provide strain-transcendent immunity in animal model systems [13–19]. One of the important differences between these animal model systems and humans is that in contrast to naive mice and ferrets, humans typically have prior immunity generated by exposure to multiple strains that have circulated in the past. Consequently, to move these vaccines to humans, it would be helpful to develop a quantitative understanding of how pre-existing immunity affects the ability of vaccines to boost antibody responses in general and the response to the stem of HA in particular.

**Fig 1:**
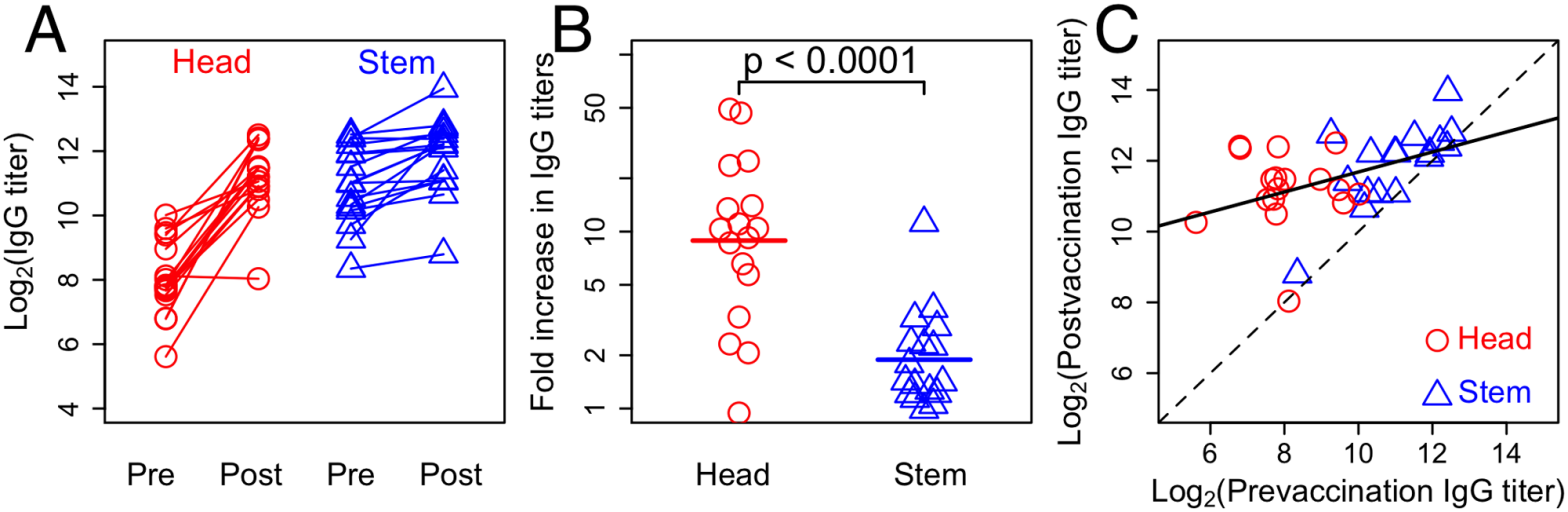
Boosting of antibodies to the head and stem epitopes of HA following vaccination with inactivated H5N1. Panel A shows IgG titers against HA head (red) and stem (blue) epitopes measured prevaccination and 30 days post-vaccination. Panel B shows the fold-increase in IgG antibody titers against HA head (red) and stem (blue) epitopes calculated from the data in panel A. Panel C shows the relationship between the pre- and post-vaccination antibody titers. In the absence of boosting, we expect the data to fall on the dashed line (slope = 1). If the degree of boosting is independent of the initial titer, boosting would result in the data falling on a line parallel to (and above) the dashed line. The solid line, representing the best fit line, has slope less than one (least squares; slope = 0.28; 95% CI = [0.090;0.476]), indicating that there is less boosting when initial antibody titers are high. Data are from [20].

In this paper we develop mathematical models to help us explore different hypotheses for how pre-existing antibody affects boosting following immunization. We define the magnitude of the boost as the fold increase in antibody following immunization. The models are confronted with reanalysis of recent data shown in Fig 1 measuring how immunization with a vaccine containing HA from novel strains of influenza boosts antibodies specific to the stem and head of HA [20]. Fig 1 shows that: prior to immunization there were on average higher levels of antibodies to the stem than the head of HA (and no individuals with very low titers to the stem); and immunization caused antibodies against stem epitopes to be boosted less than those against head epitopes (Fig 1A and 1B, *t* − *test* *p* − *value* < 0.0001 and Fig A Panels A,B in S1 Text). In fact, if we look across both epitopes, increasing pre-exposure titers led to lower boosting of responses to both epitopes (Fig 1C). Similar data was obtained following immunization with inactivated H1N1 and is shown in Fig A in S1 Text. In the H1N1 study there was more overlap between the prevaccination antibody titers to the head and stem of HA. The heterogeneity in immune responses of individuals in the population makes it hard to discern the rules which govern how pre-existing immunity affects the boosting of antibody responses following immunization from these data.

We use the models together with statistical analysis to explore three hypotheses that may explain why pre-existing antibodies reduce the boosting of the humoral immune response following immunization; in doing so, we parsimoniously account for the differences in the immune responses to head and stem epitopes. The antigen clearance model (ACM) proposes that pre-existing antibodies, which bind to epitopes on antigens, cause rapid clearance of the antigen. The reduced antigen load results in less expansion of B cells and less boosting of antibody. The Fc receptor-mediated inhibition model (FIM) proposes that pre-existing antibodies bind antigen and these antigen-antibody complexes inhibit the activation of specific B cells. The proposed mechanism involves antibody forming a complex with antigen and antigen-specific B cells recognizing these immune complexes presented via complement and Fc-receptors on follicular dendritic cells in the germinal center. The co-crosslinking of the B cell receptor (bound to the antigenic epitope) and the Fc*γ*RIIB (bound to the Fc portion of the antibody in the immune complex) leads to inhibition of B cell activation [21]. The epitope masking model (EMM) proposes, instead, that pre-existing antibodies that bind to epitopes on the current strain of HA mask these epitopes, thus inhibiting the binding and proliferation of B cells specific for the same and nearby epitopes but not B cells specific for distant epitopes. This inhibition is because the stimulation of epitope-specific B cells requires their binding to the epitope and physical constraints associated with the size of antibodies prevent B cell from binding to epitopes with attached antibody. Further expansion of specific B cells and production of antibody to epitopes that are masked by pre-existing antibody is thereby downregulated despite the continued presence of antigen.

We compare the models’ predictions regarding antibody titers against hemagglutinin’s stem and head with the aforementioned data. We find that all three models can account for features of the data shown in Fig 1. We then show that reanalysis of the data in a manner that takes into account linkage between the responses to head and stem epitopes within individuals allows us to discriminate between the models. We find that only the EMM is able to recapitulate key patterns regarding the relative boosting of responses to the head and stem epitopes within individuals. We show that this conclusion is robust to a variety of alternative model assumptions, including model expansion to multiple epitopes on the head of HA. Finally, we discuss the implications of our findings for the development of a strain-transcendent influenza vaccine.

## Results

### Formulation of the immunodynamics models

**Fig 2:**
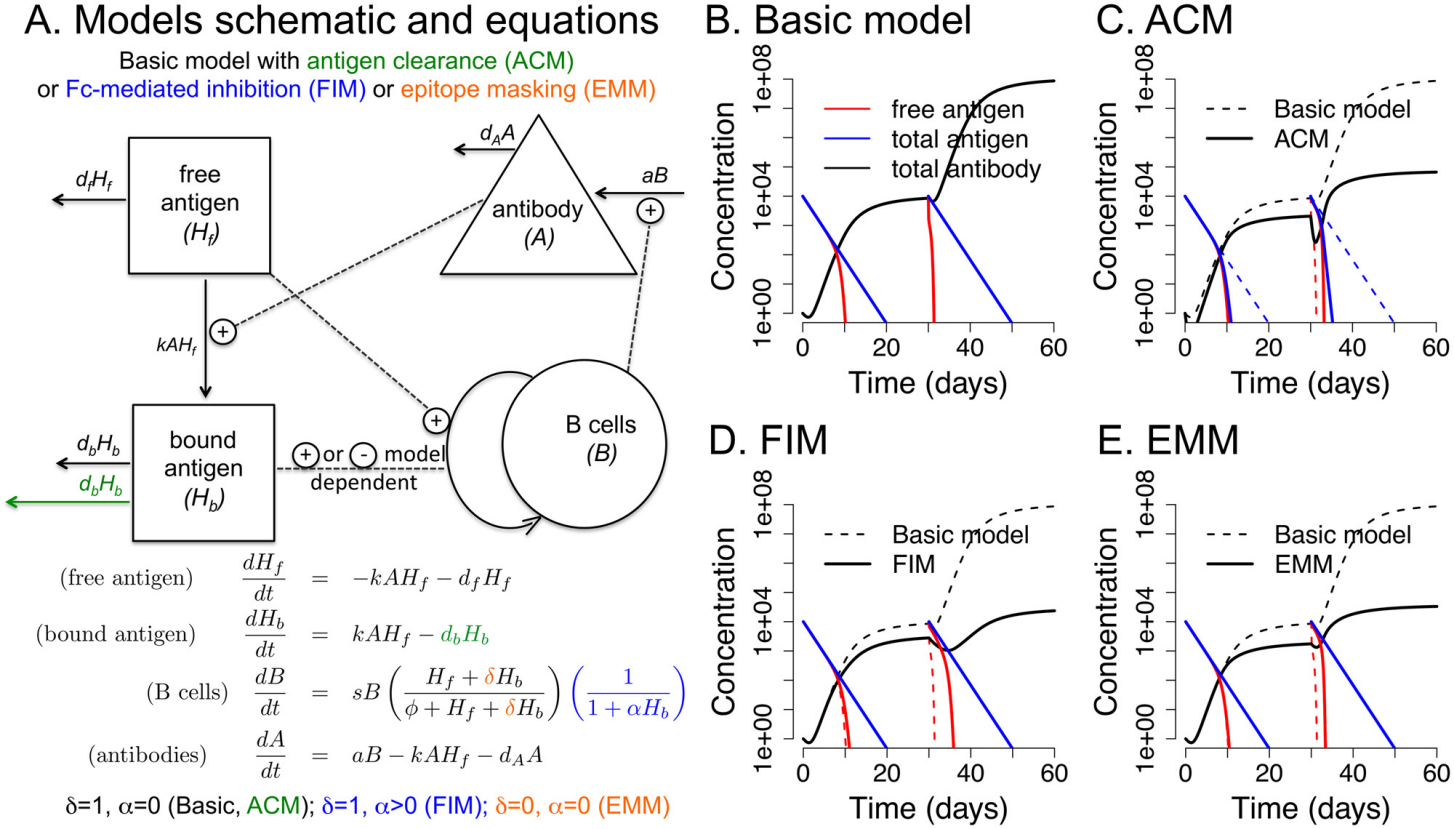
Dynamics of the immune response during primary and booster immunizations in the one-epitope model. Panel A shows a schematic and the equations for the basic one-epitope model with addition of enhanced antibody-bound antigen clearance (in green), Fc*γ*R-mediated inhibition (in blue), or epitope masking (in orange). Panels B-E show the dynamics of antigen and immune responses following primary and secondary immunization in these models. Panel B shows that in the basic model primary and secondary immunizations result in identical boosts (fold increases in antibody). Panels C, D and E show that in the ACM, FIM and EMM, respectively, the antibody generated during the primary response reduces the boosting of antibody following the second immunization. Parameters are shown in Table 1, *d*_*b*_ = 3 for the ACM, *α* = 0 for basic, ACM, EMM and *α* = 0.01 for FIM.

The modeled interactions between antigen and the immune system are depicted together with corresponding equations in Fig 2A and explained in detail in the Methods section. The objective is to use models to explore how prevaccination antibodies affect the boosting of antibody responses following immunization. The key features of the data are the measurements of antibodies to the head and stem of HA [20]. Accordingly, we use a minimal model that focuses on epitopes on the head and stem of HA and their interactions with B cells and antibodies specific for these epitopes. At this stage we have a simple B cell clonal expansion model for the generation of immune responses. Complex interactions such as differentiation of B cells and interactions with other cells such as follicular dendritic cells and T cells in germinal centers that underlie the process of clonal expansion are not explicitly included. This is because the experimental data does not include measurements of these quantities. Consequently, we use simpler models and focus on generating robust qualitative predictions. We test these predictions by confronting them with existing experimental data. In these circumstances the results of simpler models are typically more robust than those of complex models [22].

**Table 1:** Model parameters and initial values for variables.

**Model parameter**	**Symbol**	**Units**	**Value**
Rate constant for antibody binding	*k*	s^−1^day^−1^	0.01
Decay rate of free antigen	*d*_*f*_	day^−1^	0.5
Decay rate of bound antigen	*d*_*b*_	day^−1^	0.5
Max. prolif rate of B cells	*s*	day^−1^	1
Antigen for 1/2 max. prolif of B cells	*ϕ*	s	100
Antibody production rate	*a*	day^−1^	0.1
Decay rate of antibody	*d*_*A*_	day^−1^	0.1
Fc-mediated inhibition	*α*	*s* ^−1^	0 or 0.01
Extent of steric interference	*β*		0.95
**Model variable**	**Symbol**	**Units**	**Initial value**
Free antigen	*H*_*f*_	s	10^4^
Bound antigen	*H*_*b*_	s	0
B cells	B	s	1
Antibody	A	s	1

The parameter values for the simulations are the ones below unless otherwise specified in the figure legend (see Table B in S1 Text for more details). The units of antibody and B cells are re-scaled as described so at equilibrium the [*B*] = [*A*] (s = scaled units).

In the basic model, antigen stimulates clonal expansion of specific B cells, which produce antibody. Antibody production continues until all the antigen has decayed. The basic model predicts that the fold increase in antibody titers is independent of initial titers; therefore, under this model, the boost in antibody levels resulting from a second immunization is the same as that from the initial immunization. This is shown by simulations in Fig 2B.

The ACM, FIM and the EMM all predict that pre-existing antibody reduces the fold increase in antibody production following immunization, a pattern that is consistent with data ([20] and Fig 1). Thus secondary immunization with the same antigen leads to lower boosting of antibody in comparison to the primary immunization (Fig 2C and 2D). However, the underlying mechanisms of the ACM, FIM and EMM are different. In the ACM, antigen bound to antibody (*H*_*b*_) is removed from the system faster than free antigen (i.e., *d*_*b*_ > *d*_*f*_, green text in Fig 2A). Because bound antigen is removed more quickly, it has less time to stimulate B cells, resulting in less boosting (Fig 2C). In the FIM, antigen-antibody complexes (*H*_*b*_) reduce the activation of B cells as described by the parameter *α* (see additional term in blue color in equation for B cells in Fig 2A). 1/*α* is the concentration of antigen-antibody complexes that reduces the growth rate of B cells by a factor of 2. If *α* = 0 there is no Fc-mediated inhibition. In the EMM, antigen is cleared at the same rate whether bound or free (i.e., *d*_*b*_ = *d*_*f*_); however, bound antigen is unable to stimulate B cell proliferation because the sites to which the B cells would bind are sterically blocked (i.e., orange text and *δ* = 0 removes the two terms in corresponding equation for B cells in Fig 2A). This also results in less boosting upon re-exposure (Fig 2E).

### Application to influenza

#### Two-epitope models

**Fig 3:**
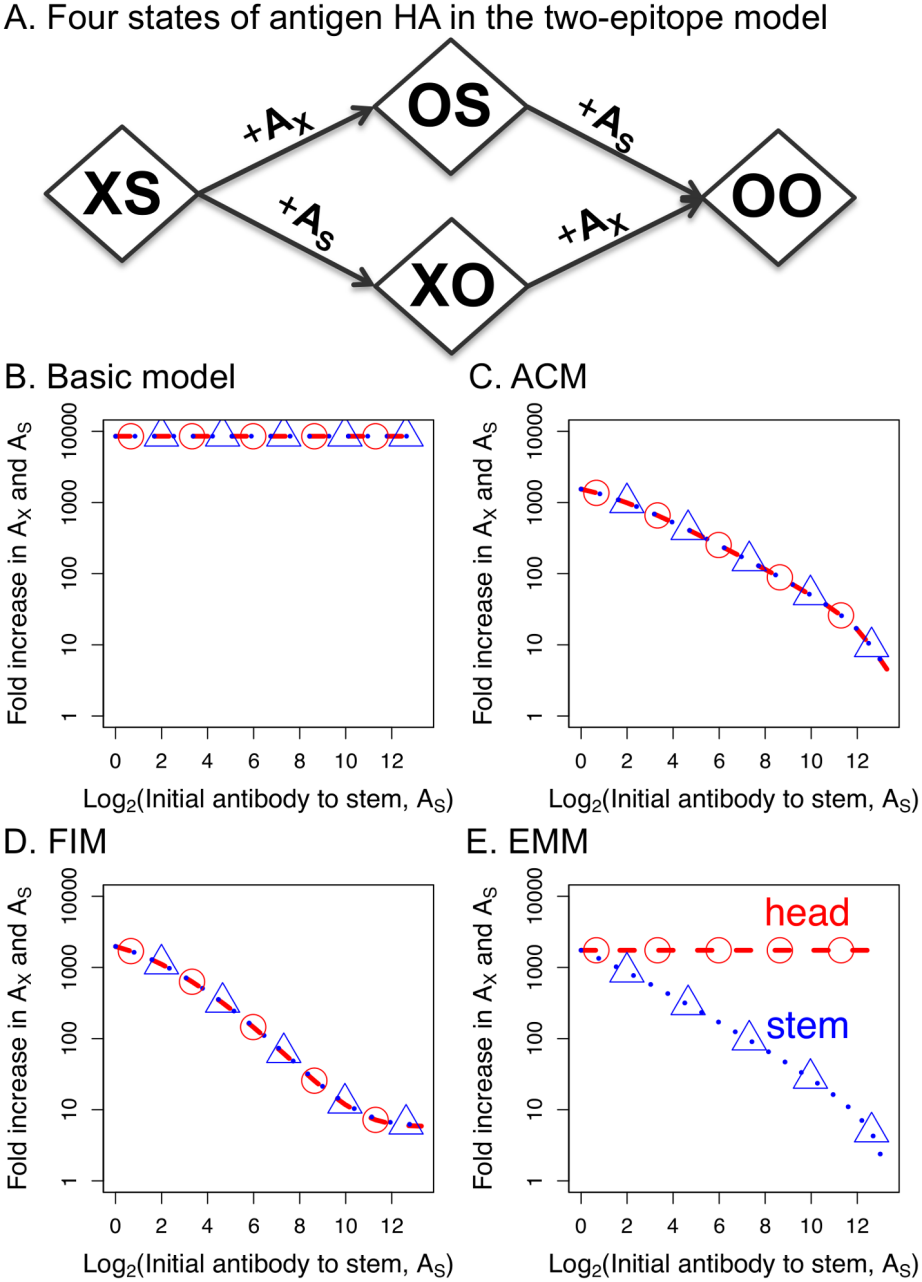
Two-epitope EMM. Panel A: A schematic for the two-epitope EMM. The HA antigen has two epitopes: *X* on the head and *S* on the stem. Binding of antibodies specific for these epitopes masks them and masked epitope is indicated by *O*. Panel B-D: We plot for the two-epitope model how pre-existing antibody to the stem of HA, *A*_*S*_, affects boosting (fold increase) in the antibody to both the head (*A*_*X*_) and the stem (*A*_*S*_) of HA following immunization. In the basic model (Panel B) boosting is independent of the level of pre-existing antibody. In the ACM (Panel C) prevaccination antibody to the stem clears the antigen and causes an equal reduction in boosting of antibodies to both the head and stem of HA. In the FIM (in the absence of epitope masking) (Panel D), prevaccination antibody rapidly binds antigen and these antigen-antibody complexes downregulate B cell proliferation to both epitopes. In the EMM (Panel E) pre-existing antibody to the stem masks only the stem epitope, thus reducing only the boosting of antibody to the stem of HA (and boosting of antibody to the head remains unaffected). Corresponding models equations are shown in Methods section. Parameters are in the Table 1. For ACM parameter *d*_*b*_ is equal 3, for FIM parameter *α* = 0.01 and *α* = 0 for other models.

We extend the one-epitope immune dynamics models to account for key aspects of influenza virus structure and evolution. The main antigenic component of the virus, HA, consists of two distinct parts, the head and the stem. Therefore, to model influenza, we develop a state-space model (Fig 3A) in which antigen can be in one of several states: *XS* corresponds to HA with no antibodies bound to either the head *X* or the stem *S* epitopes; *OS* corresponds to HA with antibody *A*_*X*_ bound to and masking the head epitope *X*; *XO* corresponds to HA with antibody *A*_*S*_ bound to and masking the stem epitope *S*; and finally *OO* corresponds to HA with antibodies *A*_*X*_ and *A*_*S*_ bound to and masking both head and stem epitopes. The symbol *O* indicates the masking of the epitope by antibody binding to that epitope. The transitions among these states occur at rates that are independent of the current state (e.g., the rate constants for *XS* + *A*_*S*_ → *XO* is the same as that for *XO* + *A*_*X*_ → *OO* etc; Fig 3A).

We combine the two-epitope state-space model with the models of immune dynamics (i.e., basic, ACM, FIM and EMM) to explore how pre-existing immunity affects the boosting of responses to both the head and stem epitopes. We consider the case of immunization of an individual with HA and a simple scenario where HA has a new head, and therefore only little (i.e. naive level) pre-existing immunity to it; however, there is some level of immunity to the conserved stem of HA (similar to the data in Fig 1). We therefore consider the host’s response by modeling the fold increase in antibody titers to head and stem epitopes in the presence of varying levels of stem antibodies and assume a naive status for B cells and antibodies to the head (Fig 3B–3E).

In the basic model, pre-existing antibodies do not affect the subsequent response, and the fold boost is unaffected by varying pre-existing antibody levels to the stem (Fig 3B, parallel with Fig 2B). In the ACM, pre-existing antibodies to the stem of HA affect both epitopes equally; head and stem antibody titers decline in tandem. This is because when antibody titers to the stem increase, binding of this antibody to the stem epitope on HA clears the entire antigen, reducing both head and stem epitopes equally (Fig 3C). In the FIM, in the absence of epitope masking we see a result similar to the ACM. Antigen bound to antibody inhibits activation of the B cells specific to both epitopes (Fig 3D). In contrast, in the EMM, high pre-existing antibody titers to the stem of HA bind to the stem and mask this epitope. This reduces the stimulation of stem-specific B cells and results in lower boosting of antibodies to the stem. However, stem antibodies have no effect on the antibody response to the head (Fig 3E). The key result which emerges is that only the EMM produces differential boosting of responses to head and stem epitopes with weaker boosting of antibodies to the stem (Fig 3B–3E).

#### Multi-epitope models and steric interference

**Fig 4:**
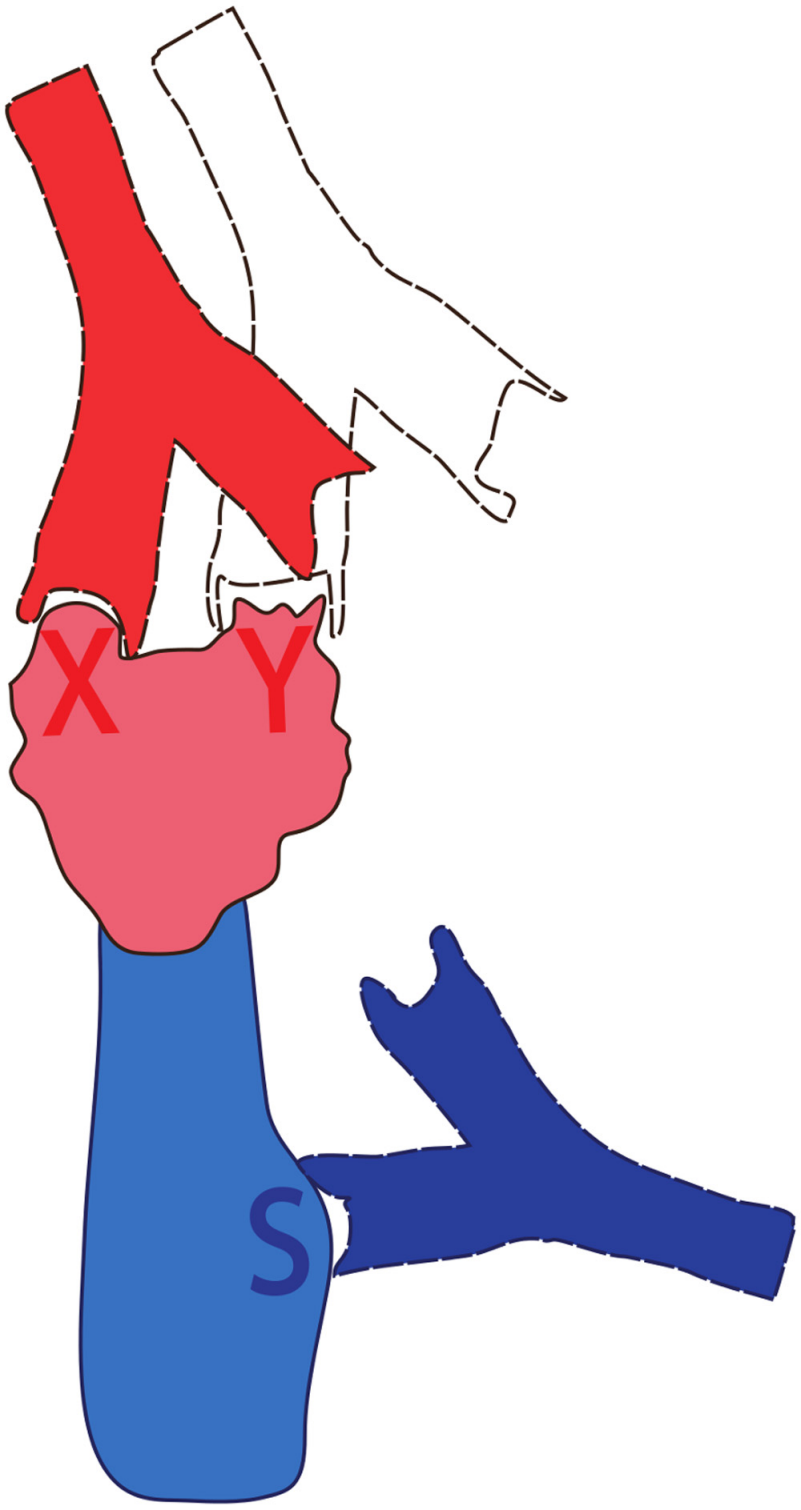
Illustration of steric interference between antibodies to the epitopes on the head of HA in the multi-epitope model. We describe antigenic drift by changing only epitope *Y* on the head of HA between the two virus strains. Antibody to *X* generated in response to a previously experienced strain sterically hinders efficient stimulation of B cells specific for the new epitope *Y*.

HA has multiple epitopes on the head and fewer epitopes on the stem. As a first step to explore the consequences of adding this complexity, we develop a multi-epitope model of HA with two epitopes on the head (i.e., *X* and *Y*) and one epitope on the stem (*S*) (Fig 4). The incorporation of multiple epitopes on the head of HA requires consideration of steric interference. Steric interference arises as a consequence of physical constraints associated with the size of antibodies and the proximity of head epitopes. Steric interference has been suggested in the context of neutralization efficiency of epitopes [23] and is supported by stoichiometric studies of binding of antibodies to the head of HA on intact virus [10, 24]. Antibody bound to a head epitope (*X* in Fig 4) not only blocks the binding of further antibody or B cells to the same epitope, it also inhibits binding to nearby epitopes on the head of HA (e.g., *Y* in Fig 4) but not to the more distant epitopes on the stem (epitope *S* in Fig 4, also see Fig B in S1 Text for details of the multi-epitope model with three epitopes and corresponding equations in Methods section).

In the multi-epitope model, antigenic drift would correspond to a change in one of the head epitopes. We assume that epitope *X* does not change between the old and new strains, whereas epitope *Y* on the new strain is novel (i.e. substantially changed in comparison with the old strain). Therefore, pre-existing antibody to *X* is fully functional, whereas little pre-existing antibody binds to the new epitope *Y*. However, steric hinderance would result in pre-existing antibody binding to *X* reducing the boosting of antibody to *Y*. When the parameter *β*, which describes the extent of steric interference approaches 1, the fold increase in the antibody to epitope *X* and *Y* becomes almost identical, significantly limiting generation of antibody to *Y* when pre-existing levels of antibody to *X* are high.

The multi-epitope models with steric interference generate dynamics and predictions similar to the two-epitope models (see Fig B in S1 Text). In particular, in the context of the multi-epitope model, the basic, ACM and FIM formulations do not allow for differences in the fold increase (boost) of antibodies specific to head and stem epitopes, whereas the EMM does. These results demonstrate that the results of our model are robust to adding more epitopes to the head of HA.

### Model discrimination

The data in Fig 1C showing that increasing the amount of pre-existing antibody reduces the extent of boosting is consistent, thus far, with all three hypotheses (i.e. the ACM, FIM and EMM). We now tackle the problem of discriminating between the ACM, FIM and EMM by identifying scenarios where the models give rise to different predictions. In what follows we simulate the responses to immunization with HA using a multi-epitope framework with three epitopes, but we note that we get the same results using a two-epitope framework.

**Fig 5:**
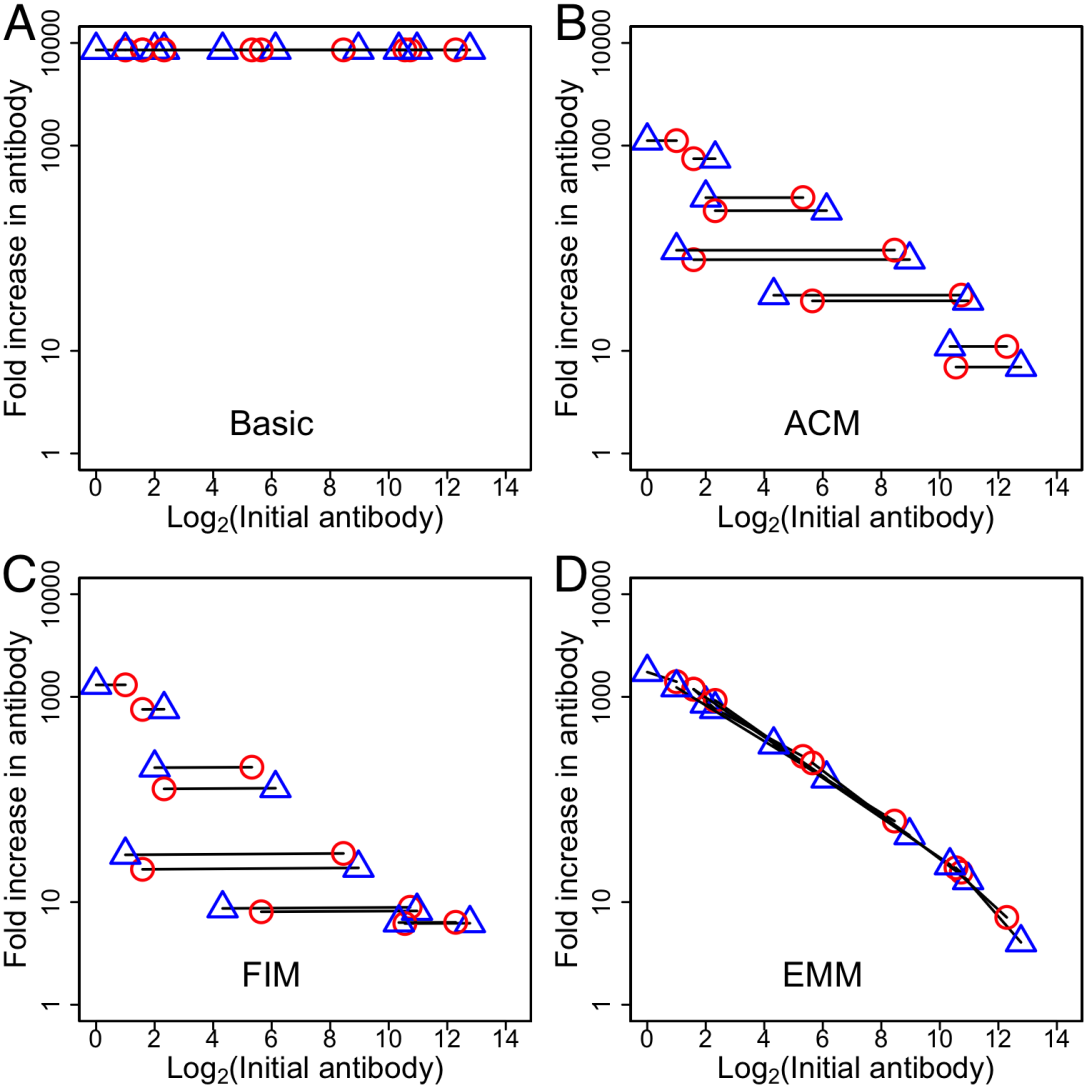
Predictions of the different models when different individuals vary in the level of pre-existing antibody to both head (red circles) and stem (blue triangles) epitopes. The basic, antigen clearance (ACM), Fc-mediated inhibition (FIM) and epitope-masking (EMM) models generate different predictions. Using a three-epitope framework we calculate how different amounts of pre-existing immunity to the head and stem of HA affect boosting of the antibody responses to these epitopes. We consider individuals with different amounts of antibody to the head and stem of HA prior to immunization. The ten different initial conditions are shown. Antibody boosting to a pair of epitopes (head epitope *X* in red and stem epitope *S* in blue) in a given individual is connected by a line. In the basic model (Panel A) boosting is independent of the level of pre-existing antibody. In the ACM (Panel B), pre-existing antibody to either epitope clears the entire antigen and thus causes an equal reduction in the boosting of responses to both head and stem epitopes (the lines for each individual are horizontal). In the FIM (Panel C), antibodies bound to antigens (antigen-antibody complexes) equally inhibit the activation of B cells specific for both head and stem epitopes. In the EMM (Panel D), pre-existing antibody binding to either the head or stem epitopes only reduces the boosting of the response to that epitope and not the other one. Other parameters as in Table 1. For ACM parameter *d*_*b*_ is equal 3, for FIM parameter *α* = 0.01 and *α* = 0 for other models.

The experimental data show boosting of antibodies to the head and stem of HA following immunization with H5N1 (Fig 1) and H1N1 (see Fig A in S1 Text) vaccines. In the previous sections we have considered the consequences of changing prevaccination immunity to one epitope (the stem epitope). Fig 5 shows that the models give different predictions when we vary the prevaccination immunity to both head and stem epitopes simultaneously. In Fig 5, we consider a number of individuals with different levels of pre-existing antibody to the head and stem of HA and plot the boosting of the responses to both head and stem; points connected by a line are from the same individual. In the basic model, boosting is independent of the level of pre-existing antibody; thus, the lines are all horizontal and at the same height. In the ACM, pre-existing antibody to either the head or stem of HA clears the entire antigen (i.e., both head and stem) and reduces the boosting to both head and stem equally. In the FIM, antigen-antibody complexes inhibit the activation and proliferation of B cells specific for both epitopes. Thus, in both the ACM and FIM the lines joining each individual are horizontal; however, in comparison with the basic model, the boost is reduced by pre-existing antibody to both head and stem. In contrast, in the EMM, antibodies to the head and stem of HA can be boosted by different amounts (Fig 5C). This is because pre-existing antibody to the stem of HA masks only the stem epitope, thus reducing the boosting of responses to the stem and not to the head (and vice versa for antibodies to the head). Consequently, the lines for all individuals fall along a diagonal with a negative slope.

**Fig 6:**
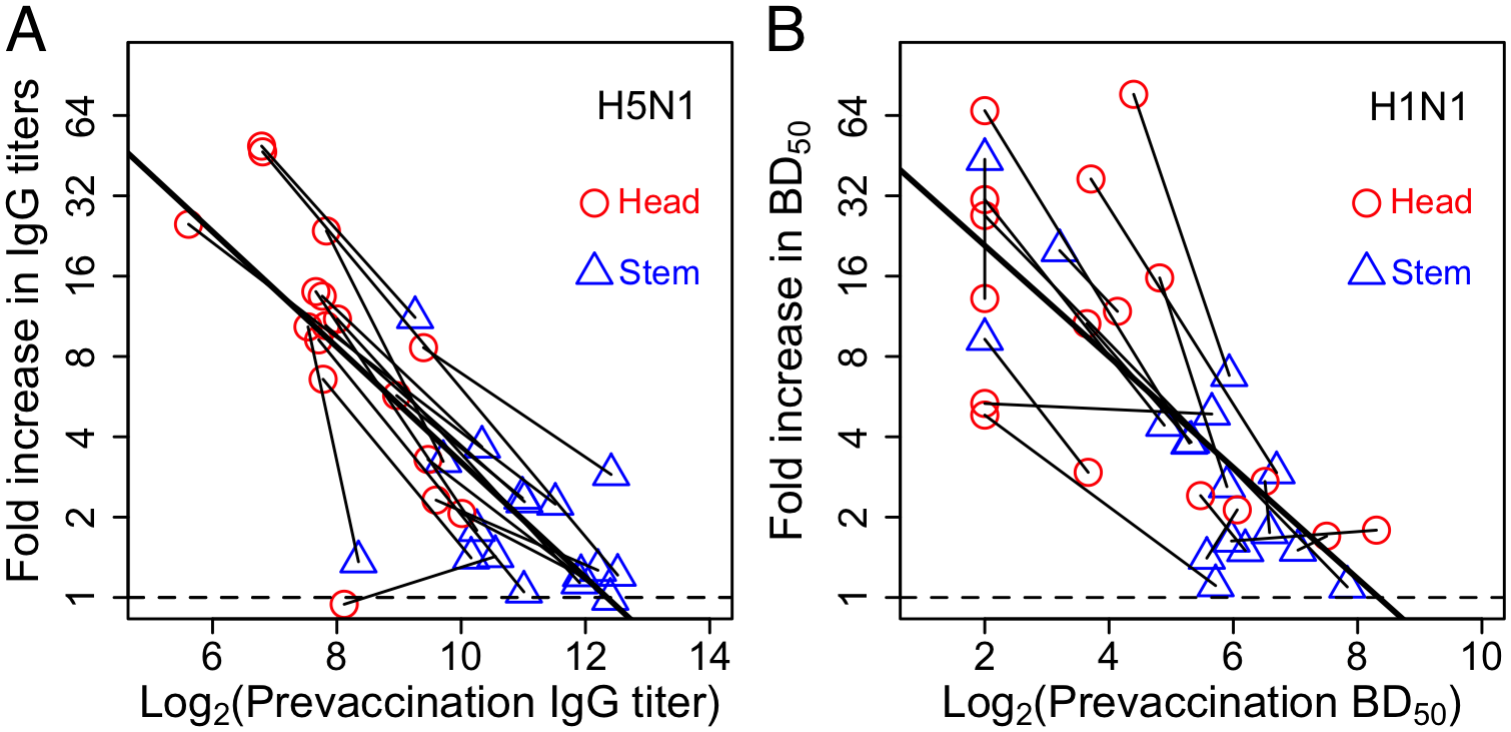
Analysis for how the fold increase in antibodies to the head and stem of HA depend on their pre-immune levels in individuals. We plot lines obtained by joining the data for head and stem for individuals vaccinated with H5N1 (Panel A) and H1N1 (Panel B) (see Tables C and D in S1 Text). We find the slope of these lines is not significantly different from an average line using all the data (thick line). This result consistent with the EMM model, but inconsistent with the ACM and FIM models which predict the slopes of the individual lines should be zero. Also see corresponding Table 2.

To discriminate between the models we compare the predictions with data from vaccine trials in humans with HAs from H5N1 or H1N1 strains (Fig 1 and Fig A in S1 Text). The assays used in the study allowed for independent measurement of stem and head antibody titers in each individual [20, 25]. The data for boosting of antibodies to the head and stem of HA such as that shown in Fig 1 were reanalyzed to generate a plot similar to that in Fig 5. The reanalyzed data is plotted in Fig 6. Clearly, the pattern observed in the data is consistent with only the EMM. The EMM predicts that low pre-existing antibody titers to epitope on the stem allow for a strong boost to that epitope regardless of whether titers to the epitopes on the head are high or low (and vice versa). In line with this prediction, the degree of boosting for a given epitope shows a significant negative correlation with the initial titers for that epitope but no significant correlation with the initial values for the other epitope (see Figs C,D and Table A in S1 Text).

We finally consider an ensemble of models that includes all possible combinations of antigen clearance, Fc-mediated inhibition and epitope masking (Fig E in S1 Text). The results are complex and indicate potentially interesting interactions between the different mechanisms of antigen clearance, Fc-mediated inhibition and epitope masking. For example, combinations that include both the FIM and EMM models (i.e. FIM+EMM and ACM+FIM+EMM) can generate slopes for individuals that are positive in comparison with the zero or negative slopes seen when we have the ACM, FIM or EMM alone. All combinations (of two or all three models) predict that the slope for each head-stem pair (slopes of individuals) will be less steep than the slope of the best fit line to the data set as a whole. This is not consistent with the observations as can be seen in Table 2 where we see that the average for the slopes of individuals is not significantly different from the slope fitted to all data points (*p* − *values* = 0.40 and 0.31 for H5N1 and H1N1, respectively). In conclusion, the data suggest that epitope masking is the key factor in reducing the boosting of antibody and that antibody-mediated antigen clearance and Fc-mediated inhibition of B cell activation play no more than a minor role.

**Table 2:** Statistical analysis of data shown in Fig 6.

Immunization	Slopes (mean with 95%CI)		p-values	
	Individual	average of all data	ACM or FIM	EMM
H5N1	-0.27 [-0.30;-0.14]	-0.22 [-.027;-0.16]	0.0003	0.40
H1N1	-0.38 [-0.72;-0.03]	-0.21 [-0.28;-0.14]	0.0338	0.310

We see that the slope of the lines obtained by joining the data for head and stem for individuals is not significantly different from an average line obtained using all the data (a result consistent with the EMM model) but significantly different from zero (the prediction of the ACM model). Note, that during H1N1 analysis we disregarded the data for one head-stem outlier with vertical slope.

### Consequences for vaccination

Epitope masking was found to be the dominant factor for explaining patterns in the data; we therefore use the EMM to predict the optimal vaccination strategy for boosting stem antibody titers. We find that increasing the dose of stem antigen used in the vaccine can help counteract the effects of epitope masking and allow for the generation of stronger antibody responses to the stem. There is a threshold level of antigen above which the response is quite strong; this level is equal to the amount of antibody present before the vaccine. Once the antigen dose in the vaccine exceeds the amount of antibody present, the free antigen successfully stimulates a boost to stem antibody titers. This is illustrated in Fig F in S1 Text where we plot how both the level of pre-existing immunity and antigen-dose affect the boosting of antibodies to a given epitope.

## Discussion

This paper uses simple mathematical models to explore how pre-existing antibody affects the generation of recall responses following immunization. We apply these models to the context of antibody responses to HA which is the main target of humoral immunity to influenza. The first step in this paper was to show that multiple models of immunodynamics, the ACM, FIM and EMM are consistent with previous observations describing how prior antibody limits the boosting of antibodies to HA upon re-exposure. In particular, we extend our previous study that described the epitope masking hypothesis [26] by showing that the clearance of antigen by antibodies (i.e., the ACM) and Fc-mediated inhibition of B cell activation (i.e., the FIM) could also explain these observations. This is because, in all three models, the presence of pre-existing antibody leads to lowering the expansion of specific B cells and less boosting.

In order to discriminate between the ACM, FIM and EMM we determine situations in which they give rise to different predictions. We find that reanalysis of the data in a manner that takes into account information previously ignored (i.e., the relative boosts to head and stem antibodies within an individual) allows us to discriminate between the two models. This discrimination is possible because the three models provide different predictions in the presence of multiple epitopes on the same antigen to which the host has differing levels of pre-existing immunity. Under the ACM, the whole antigen is affected simultaneously. Under the FIM, the antigen-antibody complexes reduce the stimulation of all antigen-specific B cells. This results in the boosting of antibodies to all the epitopes being similar in both the ACM and FIM. In the EMM, epitopes can be affected independently, and the boosting of antibodies to one epitope is not affected by antibodies to the other epitope. Comparison of these predictions with the data allows us to reject the ACM and FIM models—only the EMM is consistent with the data, and suggests that the masking of antigen epitopes by antibodies plays a key role in modulating recall responses to influenza.

We have considered how steric-interference and epitope-masking affect the generation and boosting of antibody responses. Steric interference in the binding of antibodies to epitopes on the head of HA may also have important consequences for the neutralization of live virus and this has been described in [23, 27]. The antibodies binding to non-neutralizing epitopes on the head of HA may sterically prevent neutralizing antibodies binding to nearby epitopes, and it has been suggested that HA in vaccines should be engineered to remove the non-neutralizing epitopes [27]. Our study differs from these studies in focusing on the boosting of responses rather than the neutralization of virus. Interestingly, our analysis suggests that there is little if any steric interference between the binding of antibodies to the epitopes on the head and stem of HA (see Table A in S1 Text) and that the key factor limiting the boosting of antibodies to conserved epitopes on the stem of influenza is pre-existing antibodies to these epitopes rather than antibodies to the head of HA.

The data in Fig 1A shows the response to HA from H5N1 which is not circulating in the human population, and we see that the prevaccination level of antibody to the head is much lower than that to the stem. The question is whether there are inherent differences in the head and stem that could account for the observations for the different levels of boosting to head and stem that we see in Fig 1B. We point out a few reasons against this being a major factor. First, our current models parsimoniously explain the observations. Second, if we look at the data in Fig A in S1 Text, which describes the boosting followed by vaccination with H1N1, we see that there is more overlap between the prevaccination titers to head and stem of HA and the responses to the head and stem are comparable. For example, three data points with low prevaccination titers to the stem reach post-vaccination titers similar to points having low prevaccination titer to the head of HA.

Both H5N1 and H1N1 studies show that prevaccination antibodies to the stem of HA have a minimal impact on the expansion of responses to the head of HA, and similarly prevaccination antibodies to the head do not significantly affect the boosting of antibodies to the stem of HA. This can be seen in Figs C,D and Table A in S1 Text. We note that there is some indication that increased levels of pre-existing antibodies to the head of HA might reduce the boosting of antibodies to the stem, though the *p* − *value* does not reach statistical significance (*p* − *value* = 0.137 in Fig D in S1 Text). Our modeling approach can be easily extended to consider interference between binding of antibodies to the head and stem of HA. As the relevant experimental data becomes available this should allow assessment of its role in limiting boosting of antibody to stem during scenarios of immunization with an antigenically drifted vs. shifted HA [15, 16, 28].

One could argue that if an antigen is cleaved into two separate parts then all three models (ACM, FIM and EMM) could be consistent with the independent boosting of responses to epitopes on different parts. This is unlikely to be the case for HA because the stem of HA does not maintain its native conformation in the absence of being linked to the head of HA [29, 30], so it would lead to more head than stem antigen and we would expect much higher responses to the head of HA under the same experimental settings. We have a limited dataset, but in Fig 6B we see three individuals with low preexisting antibody to the stem. The lines joining the boosting of head and stem in these individuals do not have lower slopes than the average line, indicating that there are comparable levels of stem and head epitopes.

We have used simple phenomenological models to generate qualitative predictions. This is because, in the absence of detailed information on the parameter values or sufficient data to estimate them, simpler models frequently generate more robust qualitative results than complex ones [22]. The conclusions presented here are robust to a number of variations in the model specification. First, they hold up in the context of different models of antigenic drift (i.e., two-epitope model and a three-epitope model with steric interference) and are robust to considering interactions between antibodies and B cells against multiple epitopes on the head of HA. Second, our results are not highly sensitive to parameter values within biologically reasonable ranges (see Table B in S1 Text for the choice of model parameters and Fig G in S1 Text).

The immune response to influenza vaccination is complex and not fully understood. The extensions of the models we have proposed can play an important role in furthering our understanding. An important next step is to include the differentiation of B cells during responses and reactions occurring in different locations such as the site of infection and the germinal centers of lymph nodes as well as CD4 T cell help and affinity maturation [31–33]. Doing so would require a much more complex model, and ideally would be done in conjunction with an animal model system which allows measurement of the relevant parameters and testing of various components of the model. Another extension involves going from vaccination to natural infection. This will involve incorporating resource (target cells) limitation and innate immunity [34–38] as well as T cells [39–41]. These models could be used to consider the effect of pre-existing antibodies on infection with drifted and shifted virus strains. Early studies suggested that the response of an individual would be dominated by the antibodies expanded by the first strain encountered; this was termed “original antigenic sin” [42, 43]. More recent studies have shown the evolution of the response is more complex and used the terms “antigenic seniority” and “backboosting” to describe how exposure to the current strain can lead to boosting of responses to strains that were encountered previously [44–46]. Multi-epitope models which explicitly consider the different head epitopes together with antigenic maps of changes in these epitopes may help elucidate this complex dynamics.

Another area that requires attention is the measurement of vaccine efficacy. There are many facets of vaccine efficacy; vaccines may protect against pathology (VE_*P*_), susceptibility to infection (VE_*S*_) and/or infectivity and transmission (VE_*I*_) [3, 4, 47], and estimates of influenza vaccine efficacy vary widely (e.g., the median monovalent pandemic H1N1 vaccine effectiveness in five observational studies was 69% and the range was 60% to 93% [5]). A more detailed understanding of the immune dynamics in response to influenza will be key to disentangling these complexities. Extending the EMM from vaccination to natural infection will be necessary both to further test the model and to predict its impacts on influenza epidemiology.

This study sheds light on puzzles of influenza immunity and vaccination. It elucidates rules (e.g., epitope masking) and key parameters (e.g., antigen dose) associated with the humoral immune response to influenza vaccination in non-naive hosts and may help guide the transfer of next-generation, stem-specific influenza vaccines from animal models to humans. The results suggest that explanations for differences in the antibody response to head and stem epitopes need not invoke highly different mechanisms; rather, they may result from differing pre-exposure antibody titers. Our model which is based on the results of vaccination studies in humans suggests that generating high levels of antibodies to the stem of HA (required for broad strain-transcending immunity) will involve delivery of a sufficiently high dose of antigen which overcomes the effect of epitope-masking. It would be interesting to extend models such as the ones we describe in this paper to better understand the success of immunization strategies in mice and ferrets [13–19], such as those involving nanoparticle based vaccines, that generate high levels of antibodies to the stem of HA [14, 19]. In particular, to determine whether the success of nano-particle based immunization is due to the maintenance of high levels of antigen for an extended period of time or due to other reasons such as steric accessibility of the stem in the nano-particles.

In conclusion, the confrontation of the predictions of qualitative models with a reanalysis of experimental data leads us to conclude that epitope masking is an important factor in the immune response following re-exposure to influenza’s HA. There is a clear need for a new generation of more effective and cross-protective vaccines, and understanding the key mechanisms and parameters that drive the generation of humoral immunity, such as epitope masking, prevaccination antibody titers and antigen dose, is critical.

## Methods

In the following sections we describe the models we consider in the paper.

### One-epitope model

The one-epitope model for an antibody response to an antigen includes four variables such as free antigen (*H*_*f*_), bound antigen (*H*_*b*_), B cells specific for the antigen (*B*), and the antibodies (*A*) secreted by B cells. In accord with clonal selection, B cells are stimulated and proliferate in a manner dependent on the concentration of antigen. We consider a number of scenarios. In the basic model, antigen stimulates clonal expansion of specific B cells, which produce antibody. Antibody production continues until all the antigen has decayed. In the antigen clearance model (ACM) antigen bound to antibody is removed faster than free antigen. In the Fc*γ*RIIB mediated inhibition model (FIM) antigen-antibody complexes inhibit the stimulation and proliferation of B cells. In the epitope masking model (EMM) antibody binding to an antigen masks the epitope preventing it from stimulating B cells. The equations below describe these four models (see also Fig 2A).

$$\begin{array}{ccc} (\text{free}\;\text{antigen})\;\frac{dH_{f}}{dt} & = & -kAH_{f}-d_{f}H_{f}\\ (\text{bound}\;\text{antigen})\;\frac{dH_{b}}{dt} & = & kAH_{f}-d_{f}H_{b}-d_{b}H_{b}\\ (\text{free}\;\text{antibody})\;\frac{dA}{dt} & = & aB-d_{A}A-kAH_{f}\\ (\text{B}\;\text{cells})\;\frac{dB}{dt} & = & sB\underset{\text{antigenic}\;\text{stimulation}}{\underset{︸}{\left(\frac{H_{f}+\delta H_{b}}{\phi +H_{f}+\delta H_{b}}\right)}}\underset{\text{FcR}\;\text{inhibition}}{\underset{︸}{\left(1-\frac{H_{b}}{\left(1/\alpha \right)+H_{b})}\right)}}\\ & = & sB\left(\frac{H_{f}+\delta H_{b}}{\phi +H_{f}+\delta H_{b}}\right)\left(\frac{1}{1+\alpha H_{b}}\right) \end{array}$$

For the basic model *d*_*b*_ = *d*_*f*_ = 0.5, *α* = 0, and *δ* = 1. For the ACM *d*_*f*_ = 0.5, *d*_*b*_ = 3, *α* = 0, and *δ* = 1. For the FIM *d*_*b*_ = *d*_*f*_ = 0.5, *α* = 0.01 (>0 in general), and *δ* = 1. For the EMM *d*_*b*_ = *d*_*f*_ = 0.5, *α* = 0, and *δ* = 0. The model parameters were chosen to obtain the key features of a typical antibody response. We rescaled the initial values of antibodies and B cells to unity at the naive state (prior to the first vaccination), and set *a* = *d*_*A*_, so that at equilibrium in naive or memory states we have *B* ≈ *A*. For the recall responses the initial values of antibodies and B cells were set equal to the level of preexisting immunity shown on the corresponding figures. Parameter values are specified in Table 1.

We note that B cell stimulation (by antigen) and inhibition (through Fc*γ*RIIB) are saturating functions of antigen and antigen-antibody complexes, respectively. The antigen density for half-maximal stimulation of B cells is equal to *ϕ* and the density of antigen-antibody complexes at which inhibition of B cells activation is half-maximal is equal to (1/*α*). We choose the term 1/*α* so that *α* = 0 corresponds to the absence of Fc-mediated inhibition.

### Two-epitope model

We extend one-epitope model to two-epitope model by considering an antigen with one epitope on the head of HA (*X*) and one epitope on the stem of HA (*S*). We let *B*_*X*_ and *A*_*X*_ represent B cells and antibodies specific for epitope *X* (and similarly *B*_*S*_ and *A*_*S*_ for epitope *S*). The free antigen is *H*_*XS*_, and there are three additional states for antigen: *H*_*OS*_, *H*_*XO*_ and *H*_*OO*_, representing antigen with antibodies bound to *X*, *S* or both epitopes, respectively (see schematic in Fig 3A). Parameters are the same as for the one-epitope model.

$$\begin{array}{ccc} \frac{dH_{XS}}{dt} & = & -kH_{XS}\left(A_{X}+A_{S}\right)-d_{f}H_{XS}\\ \frac{dH_{OS}}{dt} & = & kH_{XS}A_{X}-kH_{OS}A_{S}-d_{b}H_{OS}\\ \frac{dH_{XO}}{dt} & = & kH_{XS}A_{S}-kH_{XO}A_{X}-d_{b}H_{XO}\\ \frac{dH_{OO}}{dt} & = & k\left(H_{XO}A_{X}+H_{OS}A_{S}\right)-d_{b}H_{OO}\\ \frac{dB_{X}}{dt} & = & sB_{X}\left(\frac{H_{XS}+H_{XO}+\delta \left(H_{OS}+H_{OO}\right)}{\phi +H_{XS}+H_{XO}+\delta \left(H_{OS}+H_{OO}\right)}\right)\left(\frac{1}{1+\alpha (H_{XO}+\delta \left(H_{OS}+H_{OO}\right))}\right)\\ \frac{dB_{S}}{dt} & = & sB_{S}\left(\frac{H_{XS}+H_{OS}+\delta \left(H_{XO}+H_{OO}\right)}{\phi +H_{XS}+H_{OS}+\delta \left(H_{XO}+H_{OO}\right)}\right)\left(\frac{1}{1+\alpha (H_{OS}+\delta \left(H_{XO}+H_{OO}\right))}\right)\\ \frac{dA_{X}}{dt} & = & aB_{X}-k\left(H_{XS}+H_{XO}\right)A_{X}-d_{A}A_{X}\\ \frac{dA_{S}}{dt} & = & aB_{S}-k\left(H_{XS}+H_{OS}\right)A_{S}-d_{A}A_{S} \end{array}$$

### Three-epitope model with steric interference

This model considers an antigen with two epitopes (*X* and *Y*) on the head of HA and one epitope (*S*) on the stem of HA. Steric interference for the antibodies binding to the two epitopes on the head of HA is introduced into model with parameter *β*. Parameter *β* = 0 corresponds to the case of no steric interference and *β* = 1 corresponds to the case when antibody bound to one epitope on the head completely blocks binding of antibodies to the other epitope on the head. The scheme showing the transitions between the different states of an antigen with epitopes *X*, *Y* and *S* is shown in Fig B in S1 Text. The corresponding model equations are shown below. Parameters are the same as for the one-epitope model.

$$\begin{array}{ccc} \frac{dH_{XYS}}{dt} & = & -kH_{XYS}\left(A_{X}+A_{Y}+A_{S}\right)-d_{f}H_{XYS}\\ \frac{dH_{OYS}}{dt} & = & k\left(H_{XYS}A_{X}-H_{OYS}\left(\left(1-\beta \right)A_{Y}+A_{S}\right)\right)-d_{b}H_{OYS}\\ \frac{dH_{XOS}}{dt} & = & k\left(H_{XYS}A_{Y}-H_{XOS}\left(\left(1-\beta \right)A_{X}+A_{S}\right)\right)-d_{b}H_{XOS}\\ \frac{dH_{XYO}}{dt} & = & k\left(H_{XYS}A_{S}-H_{XYO}\left(A_{X}+A_{Y}\right)\right)-d_{b}H_{XYO}\\ \frac{dH_{OOS}}{dt} & = & k\left(\left(1-\beta \right)\left(H_{OYS}A_{Y}+H_{XOS}A_{X}\right)-H_{OOS}A_{S}\right)-d_{b}H_{OOS}\\ \frac{dH_{OYO}}{dt} & = & k\left(H_{OYS}A_{S}+H_{XYO}A_{X}-\left(1-\beta \right)H_{OYO}A_{Y}\right)-d_{b}H_{OYO}\\ \frac{dH_{XOO}}{dt} & = & k\left(H_{XOS}A_{S}+H_{XYO}A_{Y}-\left(1-\beta \right)H_{XOO}A_{X}\right)-d_{b}H_{XOO}\\ \frac{dH_{OOO}}{dt} & = & k\left(H_{OOS}A_{S}+\left(1-\beta \right)\left(H_{OYO}A_{Y}+H_{XOO}A_{X}\right)\right)-d_{b}H_{OOO}\\ \frac{dB_{X}}{dt} & = & sB_{X}\left(\frac{H_{XYS}+H_{XYO}+\left(1-\beta \right)\left(H_{XOS}+H_{XOO}\right)+\delta \left(H_{OYS}+H_{OYO}+H_{OOS}+H_{OOO}\right)}{\phi +H_{XYS}+H_{XYO}+\left(1-\beta \right)\left(H_{XOS}+H_{XOO}\right)+\delta \left(H_{OYS}+H_{OYO}+H_{OOS}+H_{OOO}\right)}\right)\\ & & \times \left(\frac{1}{1+\alpha (H_{XYO}+\left(1-\beta \right)\left(H_{XOS}+H_{XOO}\right)+\delta \left(H_{OYS}+H_{OYO}+H_{OOS}+H_{OOO}\right))}\right)\\ \frac{dB_{Y}}{dt} & = & sB_{Y}\left(\frac{H_{XYS}+H_{XYO}+\left(1-\beta \right)\left(H_{OYS}+H_{OYO}\right)+\delta \left(H_{XOS}+H_{XOO}+H_{OOS}+H_{OOO}\right)}{\phi +H_{XYS}+H_{XYO}+\left(1-\beta \right)\left(H_{OYS}+H_{OYO}\right)+\delta \left(H_{XOS}+H_{XOO}+H_{OOS}+H_{OOO}\right)}\right)\\ & & \times \left(\frac{1}{1+\alpha (H_{XYO}+\left(1-\beta \right)\left(H_{OYS}+H_{OYO}\right)+\delta \left(H_{XOS}+H_{XOO}+H_{OOS}+H_{OOO}\right))}\right)\\ \frac{dB_{S}}{dt} & = & sB_{S}\left(\frac{H_{XYS}+H_{OYS}+H_{XOS}+H_{OOS}+\delta \left(H_{XYO}+H_{OYO}+H_{XOO}+H_{OOO}\right)}{\phi +H_{XYS}+H_{OYS}+H_{XOS}+H_{OOS}+\delta \left(H_{XYO}+H_{OYO}+H_{XOO}+H_{OOO}\right)}\right)\\ & & \times \left(\frac{1}{1+\alpha (H_{OYS}+H_{XOS}+H_{OOS}+\delta \left(H_{XYO}+H_{OYO}+H_{XOO}+H_{OOO}\right))}\right)\\ \frac{dA_{X}}{dt} & = & aB_{X}-k\left(H_{XYS}+H_{XYO}+\left(1-\beta \right)\left(H_{XOS}+H_{XOO}\right)\right)A_{X}-d_{A}A_{X}\\ \frac{dA_{Y}}{dt} & = & aB_{Y}-k\left(H_{XYS}+H_{XYO}+\left(1-\beta \right)\left(H_{OYS}+H_{OYO}\right)\right)A_{Y}-d_{A}A_{Y}\\ \frac{dA_{S}}{dt} & = & aB_{S}-k\left(H_{XYS}+H_{OYS}+H_{XOS}+H_{OOS}\right)A_{S}-d_{A}A_{S} \end{array}$$

## Supporting Information

S1 Text **Fig A**. Boosting of antibodies to the head and stem epitopes of HA following vaccination with inactivated H1N1. Serum antibody titers against the head and stem of HA were measured by the ability of serum to block the binding of monoclonal antibodies that bind to the head and stem of HA, respectively. The BD_50_ is proportional to the serum antibody titer against the head and stem epitopes [25]. Panel A shows antibody titers in terms of BD_50_ measurements against HA head (red) and stem (blue) epitopes measured prevaccination and 30 days postvaccination. Panel B shows the fold-increase in antibody titers against HA head (red) and stem (blue) epitopes calculated from the data in panel A. Panel C shows the relationship between the pre- and postvaccination antibody titers. In the absence of boosting, we expect the data to fall on the dashed line (slope = 1). If the degree of boosting is independent of the initial titer, boosting would result in the data falling on a line parallel to (and above) the dashed line. The solid line, representing the best fit line, has slope less than one (least squares; slope = 0.31; 95% CI = [0.086,0.532]), indicating that there is less boosting when initial antibody titers are high. Data are from [20]. **Fig B**. Three-epitope model with steric interference. Panel A. Schematic for the three-epitope model with steric interference. Eight antigen states are shown. Unbound antigen (*XYS*) has three antibody binding sites, two on the head (i.e., *X* and *Y*) and one on the stem (*S*). Sites that are bound by corresponding antibody are represented with an *O*; for example, antigen with just the stem-specific antibody bound is represented as *XYO*. Panel B-E: Fold increase in antibody to the head (*X*+*Y*) and stem (*S*) as a function of preexisting humoral immunity to stem *S* (naive initial condition for humoral immunity to head epitopes *X* and *Y*) in the three-epitope versions of the basic model, ACM, FIM and EMM, respectively. Parameters are in the Table 1. For ACM parameter *d*_*b*_ is equal 3, for FIM parameter *α* = 0.01 and *α* = 0 for other models. **Fig C**. Correlation between initial antibody titers to a given epitope (on either head or stem of HA) and boosting of antibodies to the same or another epitope for H5N1 data. Analysis of H5N1 data shows that the degree of boosting of antibody to the head epitope is negatively correlated with the initial IgG titers for the same epitope (*p* − *value* = 0.0009) and not correlated with the initial IgG titers for the stem epitope (*p* − *value* = 0.34) (and vice versa for boosting of antibody to the stem epitope). See Table A in S1 Text for corresponding statistics. **Fig D**. Correlation between initial antibody titers to a given epitope (on either head or stem of HA) and boosting of antibodies to the same or another epitope for H1N1 data. Analysis of H1N1 data shows that the degree of boosting of antibody to the head epitope is negatively correlated with the initial level of antibody for the same epitope (*p* − *value* = 0.007) and not correlated with the initial values of antibody for the stem epitope (*p* − *value* = 0.98) (and vice versa for boosting of antibody to the stem epitope). Antibody titers are in terms of BD_50_ measurements [25]. See Table A in S1 Text for corresponding statistics. **Table A**. Regression analysis from Figs C and D in S1 Text. Fold increases in IgG titers to head and stem epitopes were plotted against initial IgG titers to either head or stem epitopes for H5N1 data as shown in Fig C in S1 Text and corresponding regression analysis is presented. Similar regression analysis for H1N1 data from Fig D in S1 Text is also shown. These results indicate that the dependence of boosting of antibodies to the head and stem of HA on the level of prevaccination antibodies to the head and stem epitopes, respectively, is not significantly different. It also shows that prevaccination antibody titer to the head of HA does not significantly affect boosting of responses to the stem, and vice versa. **Fig E**. Predictions of the models when different individuals vary in the level of pre-existing antibody to both head (red circles) and stem (blue triangles) epitopes. Using a three-epitope framework we calculate how different amounts of pre-existing immunity to the head and stem of HA affect boosting of the antibody responses to these epitopes in Basic model, ACM, FIM, EMM and all combinations of ACM, FIM and EMM (of two or all three models). We consider ten individuals (ten initial conditions) with different amounts of antibody to the head and stem of HA prior to immunization. **Fig F**. Effect of antigen dose and prevaccination immunity to an epitope on epitope-specific antibody boost in EMM. Prevaccination immunity reduces the boost and for high antigen doses there is approximately linear relationship between log(revaccination immunity) and log(fold increase in antibody). For a given antigen dose a threshold value of revaccination immunity exists above which there is little antibody boosting. Increasing antigen dose allows to overcome the threshold effect. **Table B**. Models parameter ranges, outcomes and robustness. Model parameters such as decay rate of antibody (*d*_*A*_) have been relatively accurately estimated *in vivo* [50] and we do not expect much variation. The maximum effective proliferation rate of B cells (*s*) was set in the range 1 ≤ *s* ≤ 2 which corresponds to division times between 1 and 0.5 days. The mean value of *a* was obtained by rescaling the concentration of antibodies so as to have *A* ≈ *B* at equilibrium, and we would expect little variation between individuals. Biological ranges for the antigen for half-maximum proliferation (*ϕ*) and decay rate of antigen (*d*_*f*_) were estimated to allow the duration of antigenic stimulation for B cells to encompass a range of 3 to 14 days. Our model is robust to the value of the rate constant for antibody binding provided *k* > 0.01 which is needed for rapid binding of antibodies to the antigen compared with the duration of the response. We note that we have rescaled the concentrations of antigen, B cells and antibodies as described in the main text and for their concentrations we use the scaled unit *s* defined as ratio of specific antibody and B cells to their value prior to vaccination. The concentration of antibodies and antigen is scaled so that *B* ≈ *A* at equilibrium. The unit of time is one day. **Table C**. Experimental Data from H5N1 vaccination study. IgG titers against HA head and stem epitopes measured pre-vaccination and 30 days post-vaccination by ELISA. For details of the study see [20]. We would like to note that this vaccination study followed both prime and boost vaccination with inactivated H5N1 avian influenza virus. We focus on the data obtained following the boost for the following reason. Individuals in the prime vaccination study were divided into two groups with first group vaccinated with Vietnam strain and second group vaccinated with Indonesia strain of H5N1. Head-specific antibodies were measured by binding to the head of HA from the Indonesia strain. It has been shown previously [48] that little cross-reacting antibody against Indonesia antigen was induced by 2 doses of Vietnam vaccine and thus we did not use the data for evaluating the fold increase in the head antibody after prime vaccination. In contrast, all individuals received a boost with the same Indonesia strain and head-specific antibodies were measured by binding to the head of HA from the same Indonesia strain. **Table D**. Experimental Data from H1N1 vaccination study. We used the data from blocking dilution (BD50) assay [25] because it measures the total specific antibody rather than ELISA measurements of IgG titers. Serum antibody titers against the head and stem of HA were measured pre-vaccination and 30 days post-vaccination by the ability of serum to block the binding of monoclonal antibodies to the head and stem of HA, respectively, and antibody titers are shown in terms of BD_50_. For details of the study see [20]. **Fig G**. Illustration of the main qualitative result for randomly selected sets of parameters in the one-epitope model. Antigen mediated clearance (ACM), Fc-mediated inhibition (FIM) and epitope masking (EMM) all reduce the magnitude of antibody boosting during secondary responses, and this is robust to changes in parameters in the one-epitope model. We used Latin hypercube sampling (LHS) [49], with the ranges of each model parameter shown in the table. Panel A, B, C and D correspond to the results of parameter variation in Basic, ACM, FIM and EMM, respectively. The antigen is shown in red and the antibody response in black. The basic dynamics is robust showing that preexisting immunity in ACM, FIM and EMM result in smaller secondary boost in comparison to primary boost. Panel E shows the summary from Panel A-D for the ratio of secondary boost versus primary boost.
(PDF)
