# Supplementary material for: Multi-epitope Models Explain How Pre-existing Antibodies Affect the Generation of Broadly Protective Responses to Influenza
Source: PLoS Pathog. 2016 Jun 23;12(6):e1005692. doi: 10.1371/journal.ppat.1005692 (PMC4918916; doi:10.1371/journal.ppat.1005692)
Supplement: S1 Text — Fig A. Boosting of antibodies to the head and stem epitopes of HA following vaccination with inactivated H1N1. Serum antibody titers against the head and stem of HA were measured by the ability of serum to block the binding of monoclonal antibodies that bind to the head and stem of HA, respectively. The BD50 is proportional to the serum antibody titer against the head and stem epitopes [25]. Panel A shows antibody titers in terms of BD50 measurements against HA head (red) and stem (blue) epitopes measured prevaccination and 30 days postvaccination. Panel B shows the fold-increase in antibody titers against HA head (red) and stem (blue) epitopes calculated from the data in panel A. Panel C shows the relationship between the pre- and postvaccination antibody titers. In the absence of boosting, we expect the data to fall on the dashed line (slope = 1). If the degree of boosting is independent of the initial titer, boosting would result in the data falling on a line parallel to (and above) the dashed line. The solid line, representing the best fit line, has slope less than one (least squares; slope = 0.31; 95% CI = [0.086,0.532]), indicating that there is less boosting when initial antibody titers are high. Data are from [20]. Fig B. Three-epitope model with steric interference. Panel A. Schematic for the three-epitope model with steric interference. Eight antigen states are shown. Unbound antigen (XYS) has three antibody binding sites, two on the head (i.e., X and Y) and one on the stem (S). Sites that are bound by corresponding antibody are represented with an O; for example, antigen with just the stem-specific antibody bound is represented as XYO. Panel B-E: Fold increase in antibody to the head (X+Y) and stem (S) as a function of preexisting humoral immunity to stem S (naive initial condition for humoral immunity to head epitopes X and Y) in the three-epitope versions of the basic model, ACM, FIM and EMM, respectively. Parameters are in the Table 1. For ACM paramete [file ppat.1005692.s001.pdf]

Figure A. Boosting of antibodies to the head and stem epitopes of HA following vaccination with inactivated H1N1. Serum antibody titers against the head and stem of HA were measured by the ability of serum to block the binding of monoclonal antibodies that bind to the head and stem of HA, respectively. The BD_{50} is proportional to the serum antibody titer against the head and stem epitopes [1]. Panel A shows antibody titers in terms of BD_{50} measurements against HA head (red) and stem (blue) epitopes measured prevaccination and 30 days postvaccination. Panel B shows the fold-increase in antibody titers against HA head (red) and stem (blue) epitopes calculated from the data in panel A. Panel C shows the relationship between the pre- and postvaccination antibody titers. In the absence of boosting, we expect the data to fall on the dashed line (slope=1). If the degree of boosting is independent of the initial titer, boosting would result in the data falling on a line parallel to (and above) the dashed line. The solid line, representing the best fit line, has slope less than one (least squares; slope = 0.31; 95% CI=[0.086,0.532]), indicating that there is less boosting when initial antibody titers are high. Data are from [2].

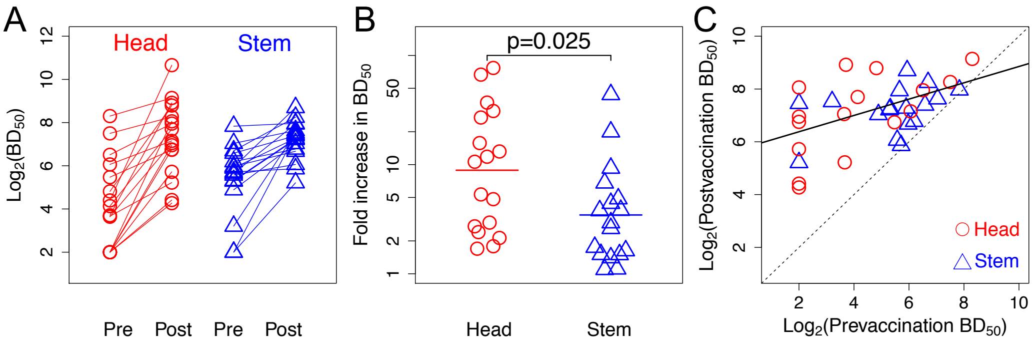

Figure B. Three-epitope model with steric interference. Panel A. Schematic for the three-epitope model with steric interference. Eight antigen states are shown. Unbound antigen (XYS) has three antibody binding sites, two on the head (i.e., X and Y) and one on the stem (S). Sites that are bound by corresponding antibody are represented with an O; for example, antigen with just the stem-specific antibody bound is represented as XYO. Panel B-E: Fold increase in antibody to the head (X+Y) and stem (S) as a function of preexisting humoral immunity to stem S (naive initial condition for humoral immunity to head epitopes X and Y) in the three-epitope versions of the basic model, ACM, FIM and EMM, respectively. Parameters are in the Table 1. For ACM parameter d_b is equal 3, for FIM parameter $\alpha = 0.01$ and $\alpha = 0$ for other models.

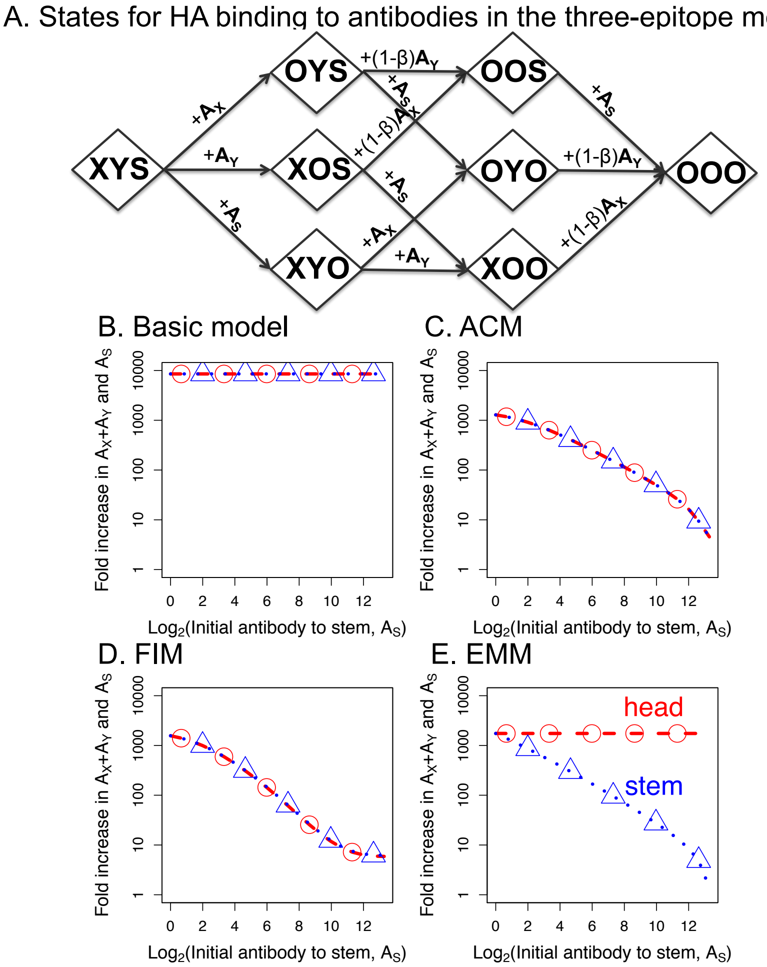

Figure C. Correlation between initial antibody titers to a given epitope (on either head or stem of HA) and boosting of antibodies to the same or another epitope for H5N1 data. Analysis of H5N1 data shows that the degree of boosting of antibody to the head epitope is negatively correlated with the initial IgG titers for the same epitope ($p - value = 0.0009$) and not correlated with the initial IgG titers for the stem epitope ($p - value = 0.34$) (and vice versa for boosting of antibody to the stem epitope). See Table A in S1 Text for corresponding statistics.

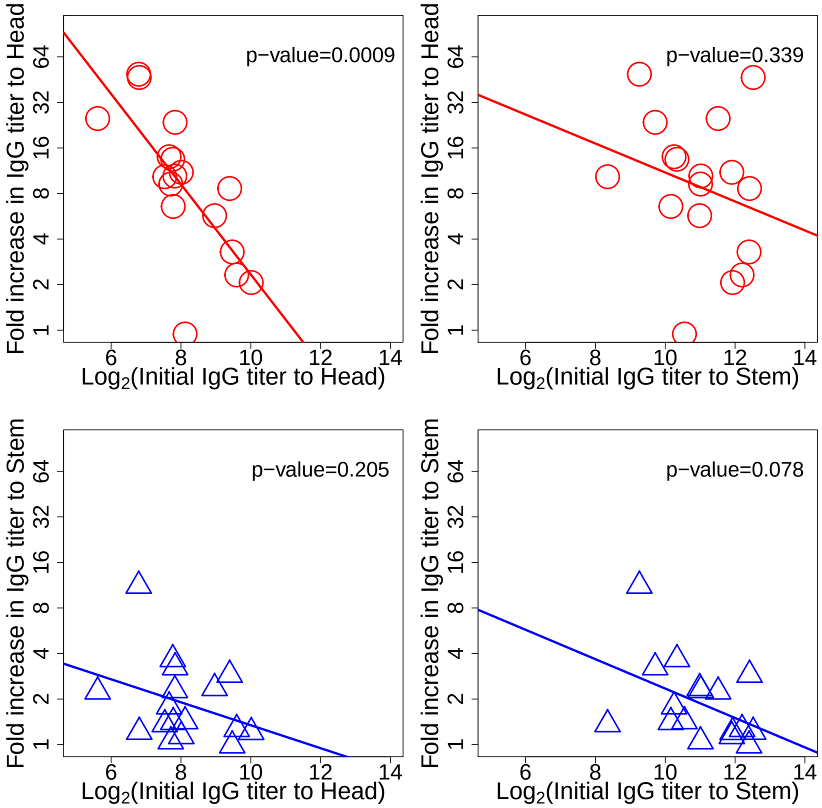

Figure D. Correlation between initial antibody titers to a given epitope (on either head or stem of HA) and boosting of antibodies to the same or another epitope for H1N1 data. Analysis of H1N1 data shows that the degree of boosting of antibody to the head epitope is negatively correlated with the initial level of antibody for the same epitope ($p-value = 0.007$) and not correlated with the initial values of antibody for the stem epitope ($p-value = 0.98$) (and vice versa for boosting of antibody to the stem epitope). Antibody titers are in terms of BD_{50} measurements [1]. See Table A in S1 Text for corresponding statistics.

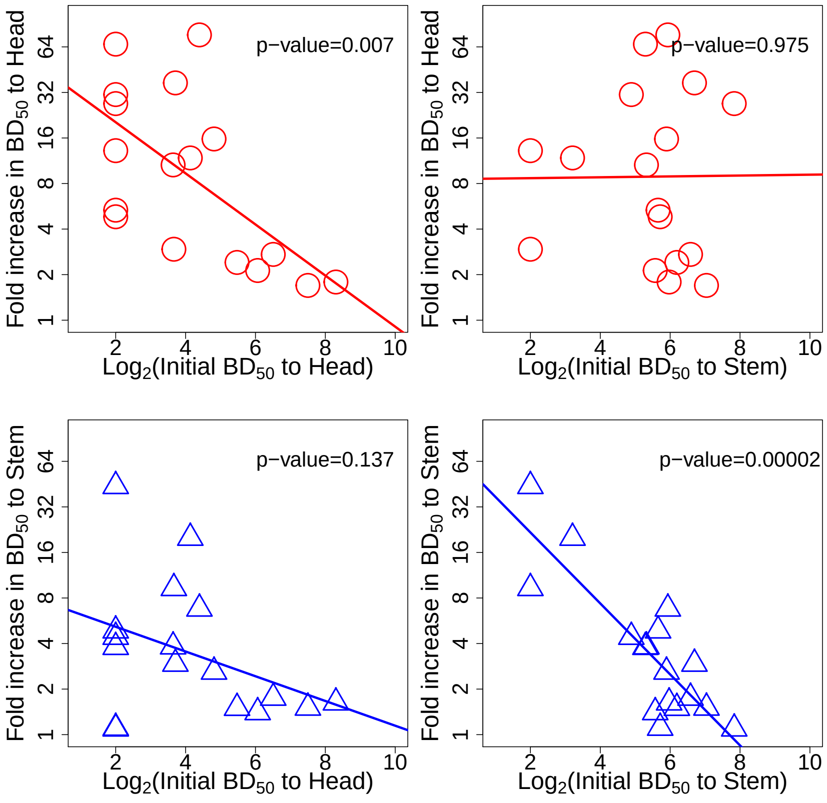

Table A. Fold increases in IgG titers to head and stem epitopes were plotted against initial IgG titers to either head or stem epitopes for H5N1 data as shown in Fig C in S1 Text and corresponding regression analysis is presented. Similar regression analysis for H1N1 data from Fig D in S1 Text is also shown. These results indicate that the dependence of boosting of antibodies to the head and stem of HA on the level of prevaccination antibodies to the head and stem epitopes, respectively, is not significantly different. It also shows that prevaccination antibody titer to the head of HA does not significantly affect boosting of responses to the stem, and vice versa.

**H5N1**	Initial IgG Titer to Head	Initial IgG Titer to Stem
Fold Increase to Head	$p - value = 0.0009$<br>slope= -0.30<br>95%CI= $[-0.45; -0.14]$	$p - value = 0.339$<br>slope= -0.10<br>95%CI= $[-0.30; 0.11]$
Fold Increase to Stem	$p - value = 0.205$<br>slope= -0.08<br>95%CI= $[-0.20; 0.05]$	$p - value = 0.078$<br>slope= -0.10<br>95%CI= $[-0.21; 0.01]$

**H1N1**	Initial BD_{50} for Head	Initial BD_{50} for Stem
Fold Increase to Head	$p - value = 0.007$<br>slope= -0.17<br>95%CI= $[-0.28; -0.05]$	$p - value = 0.975$<br>slope= 0.003<br>95%CI= $[-0.19; 0.19]$
Fold Increase to Stem	$p - value = 0.137$<br>slope= -0.08<br>95%CI= $[-0.19; 0.03]$	$p - value = 0.00002$<br>slope= -0.23<br>95%CI= $[-0.32; -0.15]$

Figure E. Predictions of the models when different individuals vary in the level of pre-existing antibody to both head (red circles) and stem (blue triangles) epitopes. Using a three-epitope framework we calculate how different amounts of pre-existing immunity to the head and stem of HA affect boosting of the antibody responses to these epitopes in Basic model, ACM, FIM, EMM and all combinations of ACM, FIM and EMM (of two or all three models). We consider ten individuals (ten initial conditions) with different amounts of antibody to the head and stem of HA prior to immunization.

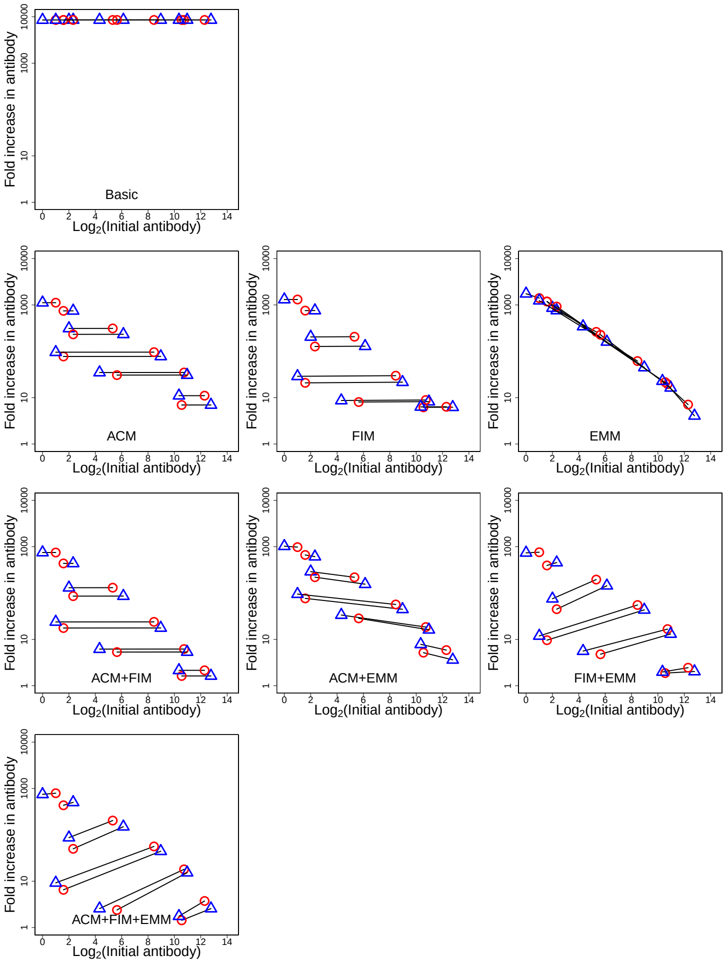

Figure F. Effect of antigen dose and prevaccination immunity to an epitope on epitope-specific antibody boost in EMM. Prevaccination immunity reduces the boost and for high antigen doses there is approximately linear relationship between log(revaccination immunity) and log(fold increase in antibody). For a given antigen dose a threshold value of prevaccination immunity exists above which there is little antibody boosting. Increasing antigen dose allows to overcome the threshold effect.

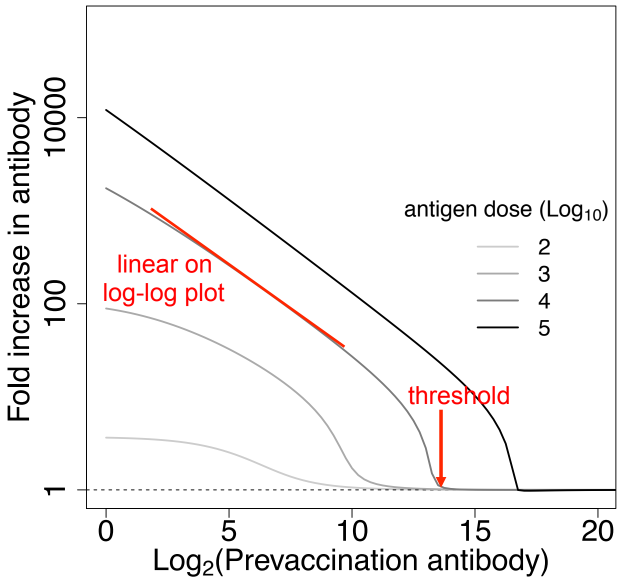

Table B. Models parameter ranges, outcomes and robustness.

Model parameter	Symbol	Units	Value	Range
Rate constant for antibody binding	k	$\mathrm{s}^{-1}\mathrm{day}^{-1}$	0.01	$0.005 - 0.05$
Decay rate of free antigen	d_f	$\mathrm{day}^{-1})$	0.5	$0.25 - 1$
Decay rate of bound antigen	d_b	day^{-1}	0.5	$0.25 - 4$
Max. prolif rate of B cells	s	day^{-1}	1	$1 - 2$
Antigen for 1/2 max. prolif of B cells	ϕ	s	10	$1 - 50$
Antibody production rate	a	day^{-1}	0.1	$0.09 - 0.11$
Decay rate of antibody	d_A	s^{-1}	0 or 0.01	$0.09 - 0.11$
Fc-mediated inhibition	α	day^{-1}	0.1	$0.001 - 0.1$
Extent of steric interference	β		0.95	0-1

Model parameters such as decay rate of antibody (d_A) have been relatively accurately estimated *in vivo* [3] and we do not expect much variation. The maximum effective proliferation rate of B cells (s) was set in the range $1 \leq s \leq 2$ which corresponds to division times between 1 and 0.5 days. The mean value of a was obtained by rescaling the concentration of antibodies so as to have $A \approx B$ at equilibrium, and we would expect little variation between individuals. Biological ranges for the antigen for half-maximum proliferation (ϕ) and decay rate of antigen (d_f) were estimated to allow the duration of antigenic stimulation for B cells to encompass a range of 3 to 14 days. Our model is robust to the value of the rate constant for antibody binding provided $k > 0.01$ which is needed for rapid binding of antibodies to the antigen compared with the duration of the response. We note that we have rescaled the concentrations of antigen, B cells and antibodies as described in the main text and for their concentrations we use the scaled unit s defined as ratio of specific antibody and B cells to their value prior to vaccination. The concentration of antibodies and antigen is scaled so that $B \approx A$ at equilibrium. The unit of time is one day.

Table C. Experimental Data from H5N1 vaccination study. IgG titers against HA head and stem epitopes measured pre-vaccination and 30 days post-vaccination by ELISA. For details of the study see [2]. We would like to note that this vaccination study followed both prime and boost vaccination with inactivated H5N1 avian influenza virus. We focus on the data obtained following the boost for the following reason. Individuals in the prime vaccination study were divided into two groups with first group vaccinated with Vietnam strain and second group vaccinated with Indonesia strain of H5N1. Head-specific antibodies were measured by binding to the head of HA from the Indonesia strain. It has been shown previously [4] that little cross-reacting antibody against Indonesia antigen was induced by 2 doses of Vietnam vaccine and thus we did not use the data for evaluating the fold increase in the head antibody after prime vaccination. In contrast, all individuals received a boost with the same Indonesia strain and head-specific antibodies were measured by binding to the head of HA from the same Indonesia strain.

**Donor**	**Head (Pre-)**	**Head (Post-)**	**Stem (Pre-)**	**Stem (Post-)**
1	227.79	5381.67	838.25	2712.21
2	672.79	5808.1	5454.81	15762.41
3	110.62	5435.3	612.65	6861.92
4	257.71	2847.77	3837.48	4357.25
5	202.5	2835.0	1219.49	2176.34
6	707.86	2327.84	5402.83	5325.0
7	277.86	262.08	1499.57	2136.52
8	48.95	1225.47	2926.304	6549.61
9	226.67	2365.75	2074.94	4741.46
10	217.47	2927.79	1291.51	4732.67
11	219.56	1442.76	1144.27	1609.74
12	499.06	2848.75	2027.68	4783.27
13	774	1793.08	4707.5	5954.67
14	1032.8	2131.88	3898.34	4710.93
15	111.88	5231.13	5867.36	7116.75
16	185.54	1916.09	325.78	443.54
17	209.0	1930.21	2061.39	2163.27

Table D. Experimental Data from H1N1 vaccination study. We used the data from blocking dilution (BD50) assay [1] because it measures the total specific antibody rather than ELISA measurements of IgG titers. Serum antibody titers against the head and stem of HA were measured pre-vaccination and 30 days post-vaccination by the ability of serum to block the binding of monoclonal antibodies to the head and stem of HA, respectively, and antibody titers are shown in terms of BD_{50}. For details of the study see [2].

**Donor**	**Head (Pre-)**	**Head (Post-)**	**Stem (Pre-)**	**Stem (Post-)**
1	66.84	142.39	47.60	66.84
2	181.16	307.50	131.53	197.90
3	12.68	37.33	4	37.13
4	44.40	106.93	73.10	109.63
5	4	108.0	228.10	249.88
6	91.07	247.83	95.88	167.81
7	315.66	564.33	62.70	102.06
8	13.07	483.56	103.65	304.32
9	12.49	132.64	39.97	152.65
10	4	52.77	4	175.35
11	4	266.81	39.17	148.43
12	21.06	1617.08	61.12	415.28
13	4	19.30	52.49	58.17
14	4	21.34	50.35	245.16
15	4	123.89	29.62	130.94
16	28.13	443.26	59.56	154.99
17	17.57	207.51	9.23	184.09

Figure G. Illustration of the main qualitative result for randomly selected sets of parameters in the one-epitope model. Antigen mediated clearance (ACM), Fc-mediated inhibition (FIM) and epitope masking (EMM) all reduce the magnitude of antibody boosting during secondary responses, and this is robust to changes in parameters in the one-epitope model. We used Latin hypercube sampling (LHS) [5], with the ranges of each model parameter shown in the table. Panel A, B, C and D correspond to the results of parameter variation in Basic, ACM, FIM and EMM, respectively. The antigen is shown in red and the antibody response in black. The basic dynamics is robust showing that preexisting immunity in ACM, FIM and EMM result in smaller secondary boost in comparison to primary boost. Panel E shows the summary from Panel A-D for the ratio of secondary boost versus primary boost.

Panel A. Basic model.

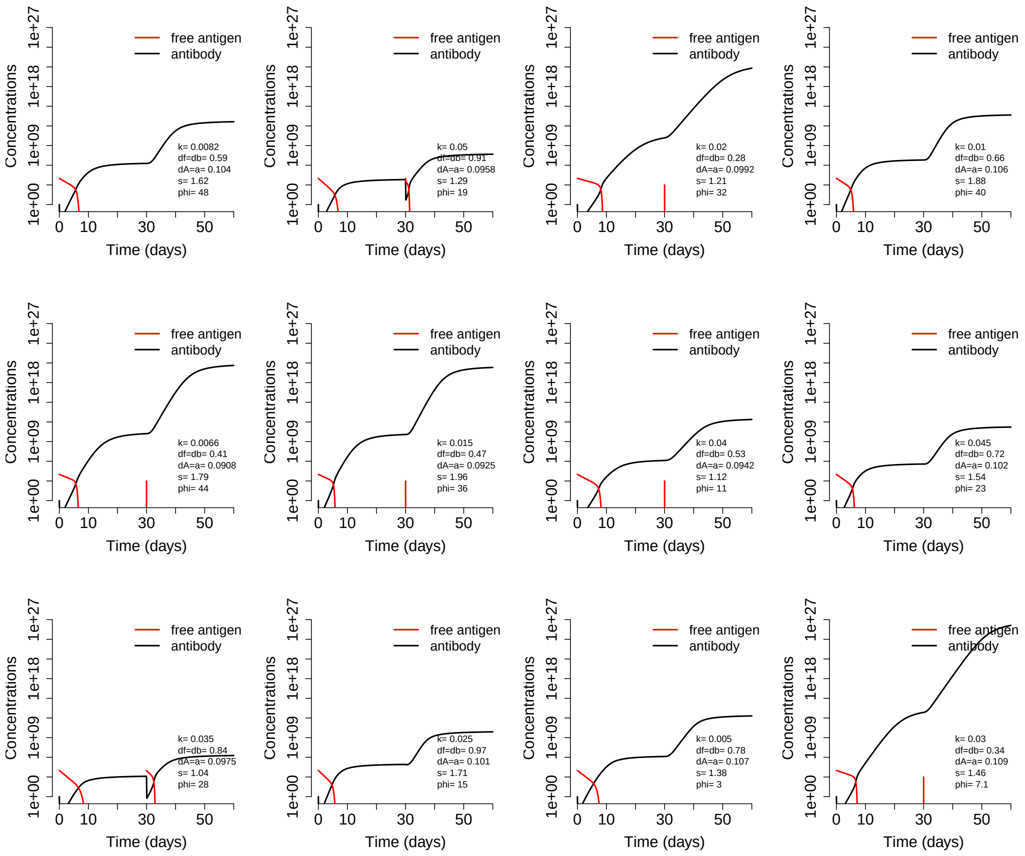

Panel B. Antigen Clearance Model.

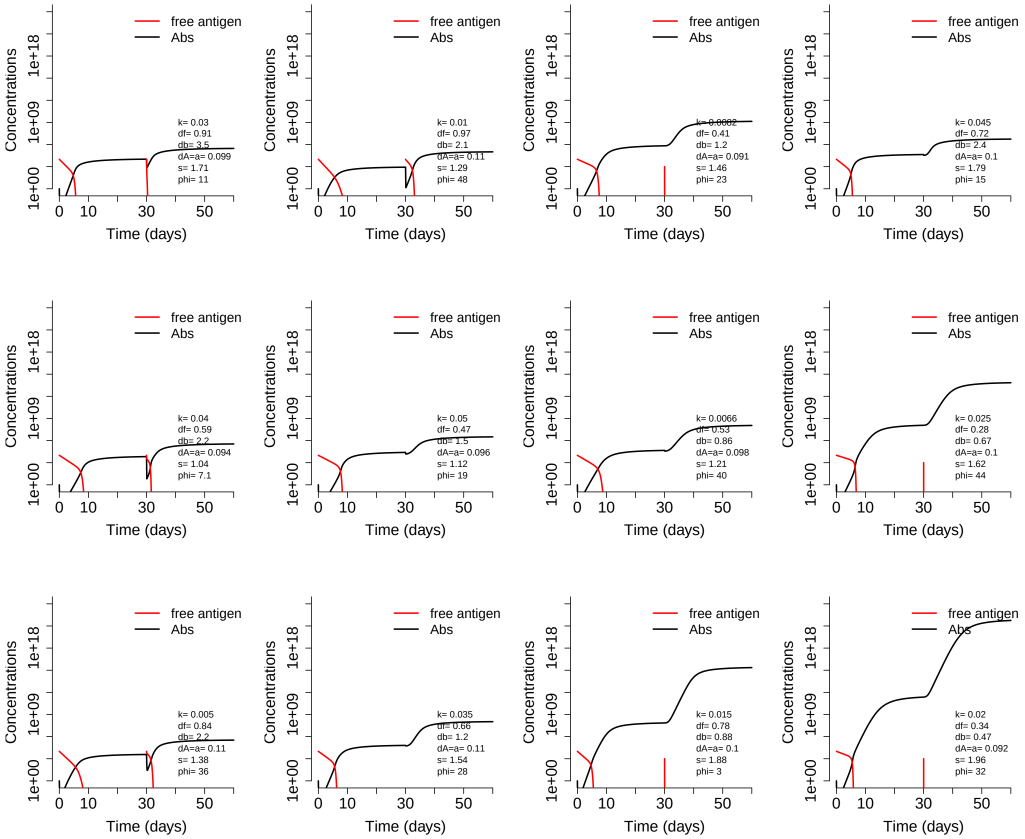

Panel C. Fc-mediated Inhibition Model.

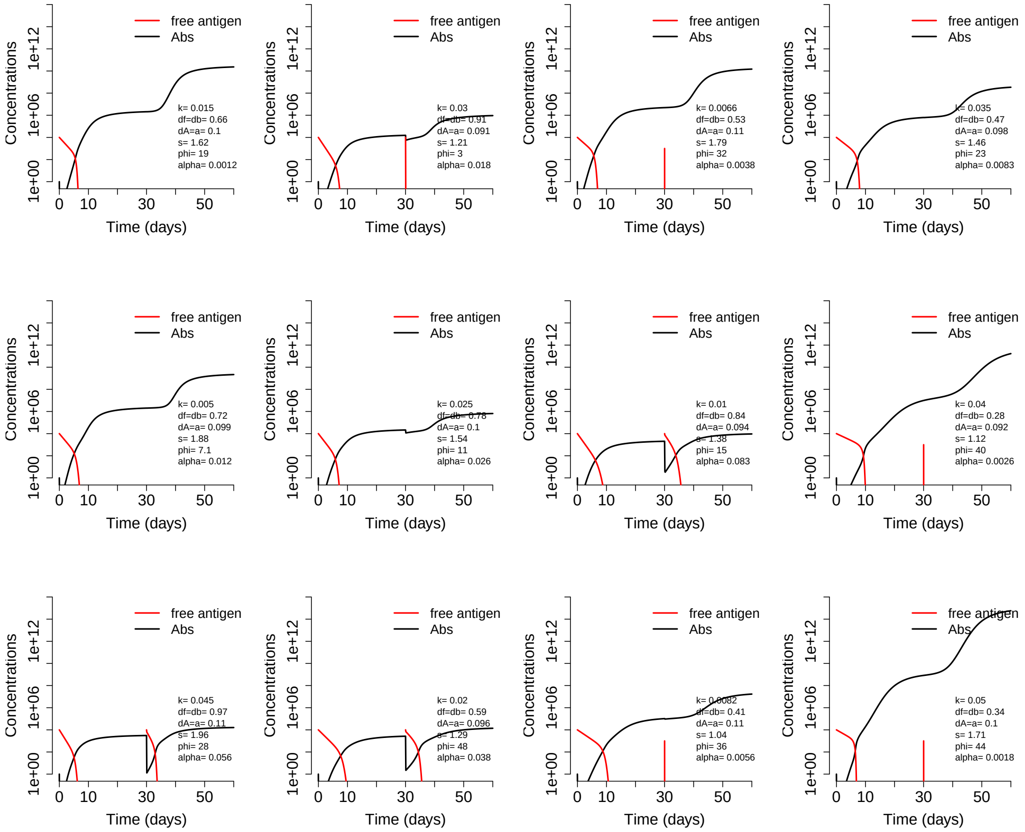

Panel D. Epitope Masking Model.

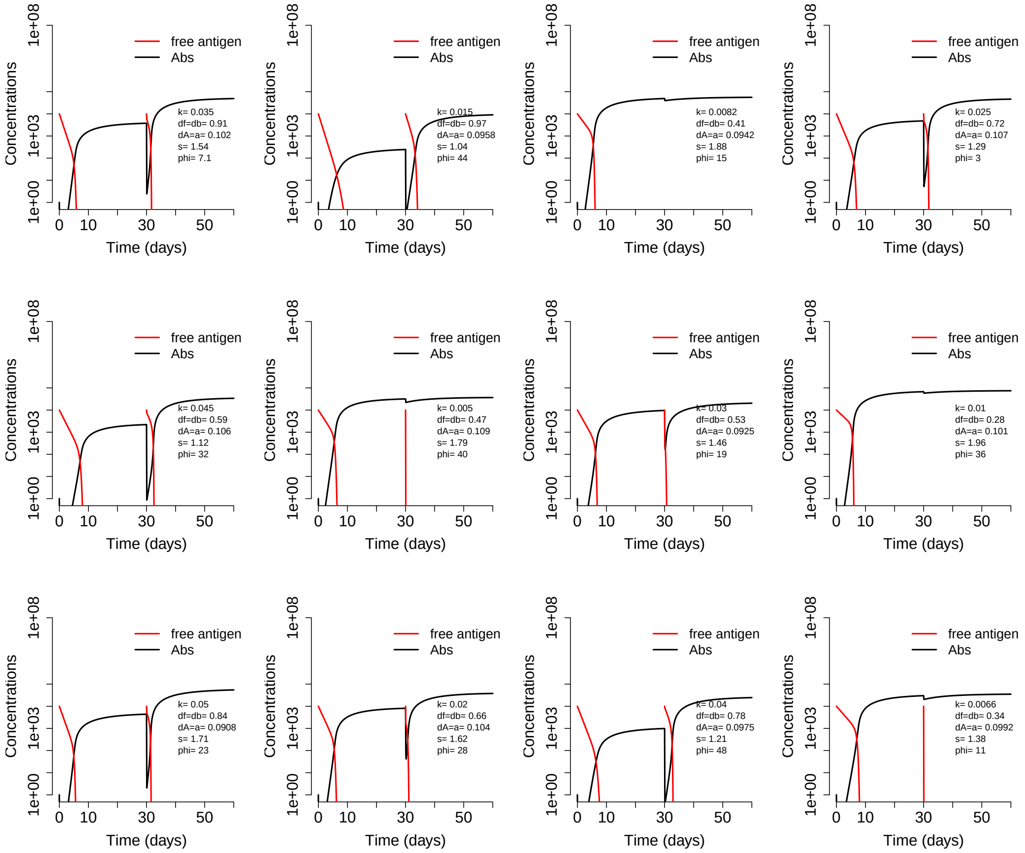

Panel E. Summary from Panels A-D.

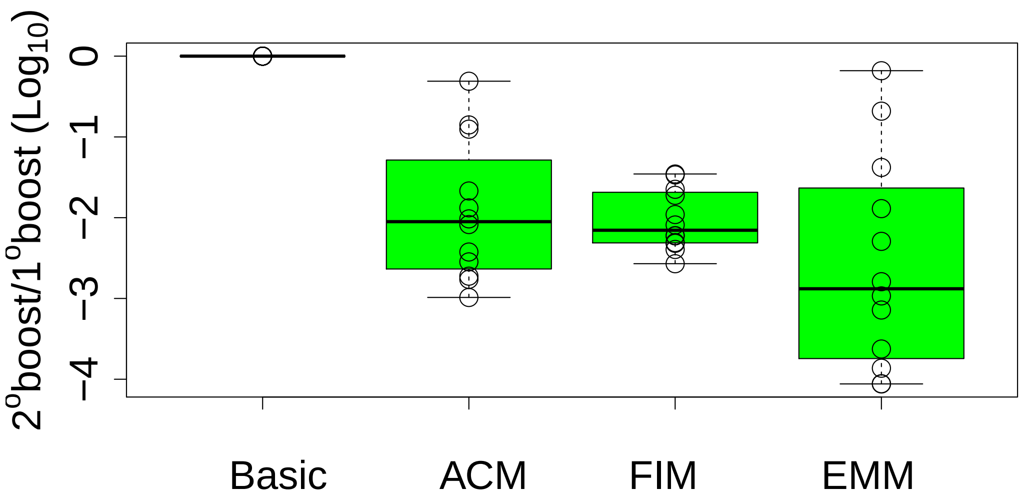

References

[1] Corti D, Suguitan AL Jr, Pinna D, Silacci C, Fernandez-Rodriguez BM, Vanzetta F, et al. Heterosubtypic neutralizing antibodies are produced by individuals immunized with a seasonal influenza vaccine. J Clin Invest. 2010 May;120(5):1663–73.

[2] Ellebedy AH, Krammer F, Li GM, Miller MS, Chiu C, Wrammert J, et al. Induction of broadly cross-reactive antibody responses to the influenza HA stem region following H5N1 vaccination in humans. Proc Natl Acad Sci U S A. 2014 Sep;111(36):13133–8.

[3] Slifka MK, Antia R, Whitmire JK, Ahmed R. Humoral immunity due to long-lived plasma cells. Immunity. 1998 Mar;8(3):363–72.

[4] Belshe RB, Frey SE, Graham I, Mulligan MJ, Edupuganti S, Jackson LA, et al. Safety and immunogenicity of influenza A H5 subunit vaccines: effect of vaccine schedule and antigenic variant. J Infect Dis. 2011 Mar;203(5):666–73.

[5] McKay MD, Beckman RJ, J CW. A Comparison of Three Methods for Selecting Values of Input Variables in the Analysis of Output from a Computer Code. Technometrics. 1979 May;21(2):239–245.
